# Computer Folding of Parallel DNA G‐Quadruplex: Hitchhiker's Guide to the Conformational Space

**DOI:** 10.1002/jcc.27535

**Published:** 2024-12-09

**Authors:** Michal Janeček, Petra Kührová, Vojtěch Mlýnský, Petr Stadlbauer, Michal Otyepka, Giovanni Bussi, Jiří Šponer, Pavel Banáš

**Affiliations:** ^1^ Department of Physical Chemistry, Faculty of Science Palacký University Olomouc Olomouc Czech Republic; ^2^ Regional Centre of Advanced Technologies and Materials, Czech Advanced Technology and Research Institute (CATRIN) Palacký University Olomouc Olomouc Czech Republic; ^3^ Institute of Biophysics of the Czech Academy of Sciences Brno Czech Republic; ^4^ IT4Innovations VŠB—Technical University of Ostrava Ostrava Czech Republic; ^5^ Scuola Internazionale Superiore di Studi Avanzati, SISSA Trieste Italy

**Keywords:** computational folding, DNA quadruplex, enhanced sampling, kinetic partitioning mechanism, metadynamics, molecular dynamics, nudged elastic band, pathCV, transition path sampling

## Abstract

Guanine quadruplexes (GQs) play crucial roles in various biological processes, and understanding their folding pathways provides insight into their stability, dynamics, and functions. This knowledge aids in designing therapeutic strategies, as GQs are potential targets for anticancer drugs and other therapeutics. Although experimental and theoretical techniques have provided valuable insights into different stages of the GQ folding, the structural complexity of GQs poses significant challenges, and our understanding remains incomplete. This study introduces a novel computational protocol for folding an entire GQ from single‐strand conformation to its native state. By combining two complementary enhanced sampling techniques, we were able to model folding pathways, encompassing a diverse range of intermediates. Although our investigation of the GQ free energy surface (FES) is focused solely on the folding of the all‐*anti* parallel GQ topology, this protocol has the potential to be adapted for the folding of systems with more complex folding landscapes.

## Graphical abstract

This study presents a novel computational protocol for folding guanine quadruplexes (GQs) from single‐strand conformation to their native state. Utilizing two enhanced sampling techniques, the protocol models multichannel folding pathways, elucidating diverse intermediates and mechanisms. Despite its focus on the all‐anti parallel GQ topology, this approach significantly enhances our understanding of GQ stability and dynamics and can be adapted for more complex folding systems.

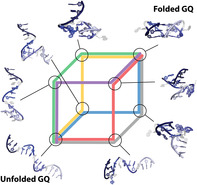

## 1 Introduction

Guanine‐rich DNA sequences are capable of forming non‐canonical four‐stranded secondary structures called G‐quadruplexes (GQs) [1, 2, 3, 4, 5]. They predominate in gene promoters or telomeres, where they play a role in key biological functions such as maintaining genome integrity, transcription, replication, or epigenetic regulation [6, 7, 8, 9, 10, 11, 12, 13, 14, 15, 16, 17, 18]. GQ formation is involved in the development of genomic diseases such as some cancers or neuropathologies [19, 20, 21] and in viral infections [22, 23], making them attractive pharmacological targets. They also find applications in nanotechnology, biosensing or biocatalysis [24, 25].

GQs consist of stacked G‐quartets, which are planar arrays of four guanines held together by *cis* Watson–Crick/Hoogsteen base pairs [26]. A channel running through the center of the GQ binds cations, which are essential for GQ stability. The G‐strands in GQs can be mutually parallel or antiparallel and they can be connected by loops that can be propeller (double‐chain reversal), diagonal, lateral (edgewise), or V‐shaped [27]. Thus, GQs can adopt a wide variety of topologies, depending on different combinations of strand direction and loop types [5]. Additional diversity arises from guanine's ability to adopt the *syn* or *anti* glycosidic conformation. Within a single G‐quartet, guanines in parallel strands require the same glycosidic bond orientation (either *syn*‐*syn* or *anti*‐*anti*), while in antiparallel strands they require opposite conformations (*syn*‐*anti*) [5]. The thermodynamic stability of different types of topologies for a given sequence is determined by the number of quartets in a given GQ, the content of *syn*‐ and *anti*‐oriented guanines, the length and composition of the loops [3], or the nature and concentration of stabilizing cations [28, 29].

Depending on the exact sequence, GQ folding times range from milliseconds up to even years in the most extreme cases [30, 31, 32, 33, 34, 35]. Therefore, the species active in cellular processes could be the GQs and the folding intermediates that are kinetically accessible on biologically relevant timescales. Alterations causing excessive stabilization or destabilization of such GQs, induced by ligands or mutations, could potentially lead to pathological effects [36].

The structural polymorphism, that is, availability of multiple metastable GQ topologies in conjunction with the sheer number of possible *syn*‐*anti* combinations, means that the GQ folding follows the kinetic partitioning mechanism: [31, 37, 38] the free‐energy landscape resembles a hypersurface with several minima corresponding to GQ folds and includes misfolded GQ structures. The initial folding to kinetically accessible GQ folds and misfolded structures is followed by slow transitions between large number of thermodynamic minima adjusting the final equilibrium population of the thermodynamically most stable GQs that account for the overall slowness and complexity of the process. The transitions between (mis)folded states are most likely to proceed via less organized and structurally fluxional intermediates. Such short‐lived species are thought to include be G‐triplexes, G‐hairpins, their cross‐like variants, or coil‐like structures [39, 40, 41, 42, 43, 44, 45, 46]. Kinetic partitioning has been demonstrated, for example, for the well‐studied human telomeric sequence (GGGTTA)_n_: convincing NMR [31, 47], sm‐FRET [33, 48, 49, 50, 51, 52] or mass spectrometry experiments [53] have shown competition between a couple of GQ folds, including misfolded ones. Unfortunately, the characterization of the less stable—and thus less populated—intermediates is beyond both the temporal and spatial resolution of these techniques.

Theoretical calculations provide a way to complement the experiments. Molecular dynamics (MD) simulations have been employed numerous times to investigate aspects that are elusive for the experimental approaches. Given the complexity of the entire folding landscape, it is common to study smaller regions of the conformational space. For example, standard MD simulations and more advanced replica‐exchange MD simulations on shorter oligonucleotides have shown that the antiparallel G‐hairpins and G‐triplexes can be populated, but their lifetimes are likely to be short [54, 55]. Parallel G‐hairpins and G‐triplexes were found even less stable, preferring geometries with crossed strands instead of fully Hoogsteen‐paired structure [56, 57]. Standard MD [46, 57, 58] and well‐tempered metadynamics simulations (metaD) [29] have suggested that the late‐stages of folding of parallel‐stranded GQs include slip‐stranded structures (i.e., GQs with fewer quartets than the native fold). There have also been studies starting from the folded state, in which a native GQ is disrupted, various intermediates are observed and then their refolding is often attempted; steered MD simulations [59, 60, 61, 62, 63, 64, 65], no‐salt simulations [66], or replica‐exchange MD simulations [67], sometimes combined with Markov State Modeling (MSM) [68, 69, 70] are typically employed in this type of approach. Results from these studies have often been combined to propose rather simplified pathways leading from a straight single‐stranded oligonucleotide to various GQ topologies via a few intermediates. Unfortunately, since the results come from simulations of somehow preselected regions of the conformational space, creation of the pathways sequentially spanning these regions is inevitably biased by the choice of the authors. Furthermore, such an approach usually neglects the transitions between GQ topologies (perhaps except for the simplest known stable GQ, the thrombin‐binding aptamer, 15‐TBA, for which there are hardly any misfolded GQ topologies).

With the gradual increase in computational power and advancements in computational techniques, attempts to fold the entire GQ‐forming sequence from scratch have emerged. Coarse‐grained (CG) models, which reduce every nucleotide into a few particles, have been first used to demonstrate interconversion between known and hypothetical human telomeric GQ (mis)folded states [71]. Then, complete folding of the telomeric hybrid GQ topologies using a hybrid model [72], later complemented by all‐atom MD simulations with MSM [73], was achieved. Its RNA counterpart, TERRA GQ, has also been folded using a CG model [74]. However, CG models often suffer from their simplified nature, making all‐atom simulations the gold standard. 15‐TBA was the first GQ structure to be completely folded at the all‐atom level [75]. Most recently, multicanonical and collective variable (CV)‐based on‐the‐fly probability enhanced sampling was used to follow folding and interconversions between various human telomeric GQ topologies [76]. Similarly, several folding pathways of a parallel‐stranded three‐quartet GQ, demonstrating the multiple‐pathway nature of the process, were described using solute tempering combined with well‐tempered metadynamics (ST‐metaD) simulations [45].

Despite significant effort, existing computational methods struggle to comprehensively capture this complex folding process at the atomic level. Although the state‐of‐the‐art enhanced sampling techniques like MetaD with path collective variables (pathCV) hold promise for elucidating the folding pathway between different conformational states, they encounter challenges when applied to large and structurally intricate systems like GQs. To gain further insights into the folding process of GQs, here we have investigated the folding mechanism of the 15‐nt DNA GQ d[(GGGA)_3_GGG] using a combination of partial nudged elastic band (NEB) method [77], well‐tempered MetaD [78], and replica exchange with solute tempering (REST2) [79] methods in explicit solvent. Given that the single nucleotide spacers between G‐tracts can essentially only form the propeller loops, the only GQ topology accessible for this sequence is the parallel topology [80, 81, 82]. We also restricted the free energy hypersurface by constraining all nucleotides in the *anti*‐orientation to further prevent the formation of kinetically stable antiparallel structures. The folding of the GQ was analyzed along the previously proposed general folding mechanism from G‐hairpin via G‐triplex to GQ. We found that consecutive guanines tend to stack into building blocks, which then form various intermediates with crossed strands; Hoogsteen‐pairing is gradually built up and eventually slip‐stranded GQs emerge. The final step is strand‐slippage, leading to the native GQ. Our results also show that the folded state is stable in our simulations only thanks to the presence of general hydrogen bond fix (gHBfix), indicating an imbalance in the original force field. The proposed procedure and results obtained in this work may help to better understand the formation of the parallel GQ and can be applied to the folding of other complex systems.

## 2 Methods

### 2.1 Starting Structures and Simulation Setup

The sequences used in this study were d(GGGAGGG), d[(GGGA)_2_GGG], and d[(GGGA)_3_GGG], denoted as 7‐mer, 11‐mer, and 15‐mer, respectively. Initial topologies and coordinates were generated using the tLEaP module of the AMBER18 software package [83]. Each structure was placed in a rectangular solvation box using the SPC/E [84] water model, ensuring minimum distance of 10 Å between the solute and the box walls. This resulted in the addition of 4145 water molecules and box size of approximately ~ 55 × 55 × 55 Å^3^ for the 7‐mer, 8047 water molecules and ~ 68 × 68 × 68 Å^3^ box for the 11‐mer, and 15,248 water molecules and ~ 82 × 82 × 82 Å^3^ box for the 15‐mer. Simulations were conducted at ~150 mM NaCl concentration using the Dang [85] ion parameters. The AMBER topologies and coordinates were subsequently converted to a GROMACS compatible format using ACPYPE tool [86].

The initial structures for the NEB simulations comprised two sets of states: endpoint structures and intermediate structures. The endpoint structures were created as follows: (i) the single‐stranded conformations were generated using Nucleic Acid Builder (AMBER14), (ii) the fully folded states of the 7‐mer, 11‐mer, and 15‐mer were obtained from the published NMR structure (PDB ID: 2LEE; the loop cytosine was replaced by adenine) [87], and (iii) partially folded 11‐mer and 15‐mer were prepared by combining a single‐stranded DNA segment with the folded hairpin or triplex, respectively (see Supporting Information S1 for details). The endpoints were connected via intermediate structures that describe the putative folding pathway. The folding pathway of the 7‐mer was adopted from our previous study [57], while the folding pathways of the 11‐mer and 15‐mer were modeled based on the NEB‐optimized folding pathway of the 7‐mer (see Supporting Information S1).

All explicit‐solvent simulations were performed with the AMBER DNA parmbsc0 [88, 89] force field with the χ_OL4_ [89] and εζ_OL1_ [90] modifications. To enhance the stability of DNA —NH⋯N— and —NH⋯O—H—bonds, a 0.5 kcal mol^−1^ increase was applied using a general external potential to tune H—bond interactions (gHBfix) [91, 92]. The gHBfix potential was originally developed to enhance stability of base pairing in RNAs [91, 92, 93, 94]. Latter, we showed that the same modification is vital also for DNA systems [45, 95]. This adjustment was implemented in all simulations except the NEB simulation of 7‐mer, where structure‐specific HBfix was selectively applied to all native —NH⋯N— and —NH⋯O— base–base interactions on a structure‐specific basis [91, 92]. Furthermore, during NEB and MetaD simulations, the χ dihedrals of all guanosines were restrained to the *anti‐*conformation using harmonic restraints with a force constant of 10 kcal mol^−1^ rad^−2^ biasing the χ dihedrals above 0° and below −180°, so that the region between 0° and 180° was penalized. For a comprehensive overview of all performed simulations, refer to Tables S1, S5, and S10 in Supporting Information S1.

### 2.2 Nudged Elastic Band Simulations

The NEB simulations for both the 7‐mer and 15‐mer utilized 40 replicas, while the NEB simulations for the 11‐mer employed either 36 or 40 replicas (see Tables S1, S5, and S10 in the Supporting Information S1 for details). All simulations were performed using the partial‐NEB method [77] with the revised tangent definition according to Henkelman and Jónsson to prevent kinks in the transition path [96]. The MPI version [97] of pmemd.cuda from the AMBER18 [83] program package for GPU acceleration was employed. The Langevin thermostat with collision frequency of 100 ps^−1^ was employed, as recommended in previous studies [97, 98]. The NEB forces were applied to all DNA solute atoms using a fixed spring constant of 5 kcal∙mol^−1^ Å^−2^. The same mask of DNA solute atoms was used for RMS fitting the neighboring images in order to remove translational and rotational motions. Initially, a 100‐ps‐long simulation was performed to heat the system from 10 K up to 298.16 K. Subsequently, all NEB simulations were conducted under NVT conditions at 298.16 K, and SHAKE algorithm combined with hydrogen mass repartitioning approach and 4 fs integration timestep, similarly as in classical MD simulation setting (see Supporting Information S1 for details). The simulations were performed on a 200 ns time scale, except for the initial 7‐mer NEB simulation, which was extended to 490 ns to assess convergence (see Figure S4).

### 2.3 Solute‐Tempering With Metadynamics

Well‐tempered MetaD in conjunction with the REST2 method, known as solute tempering with metadynamics (ST‐MetaD) [99], was employed to explore specific regions of conformational space of the studied systems. ST‐MetaD simulations were performed using the GPU accelerated version of Gromacs 2018.6 [100, 101] incorporating plumed 2.5.1 [102] and MPI support. The simulations were performed under constant volume (NVT conditions), with the Nosé‐Hoover thermostat [103] maintained at 298 K and a coupling time constant of 0.5 ps.

**FIGURE 1:**
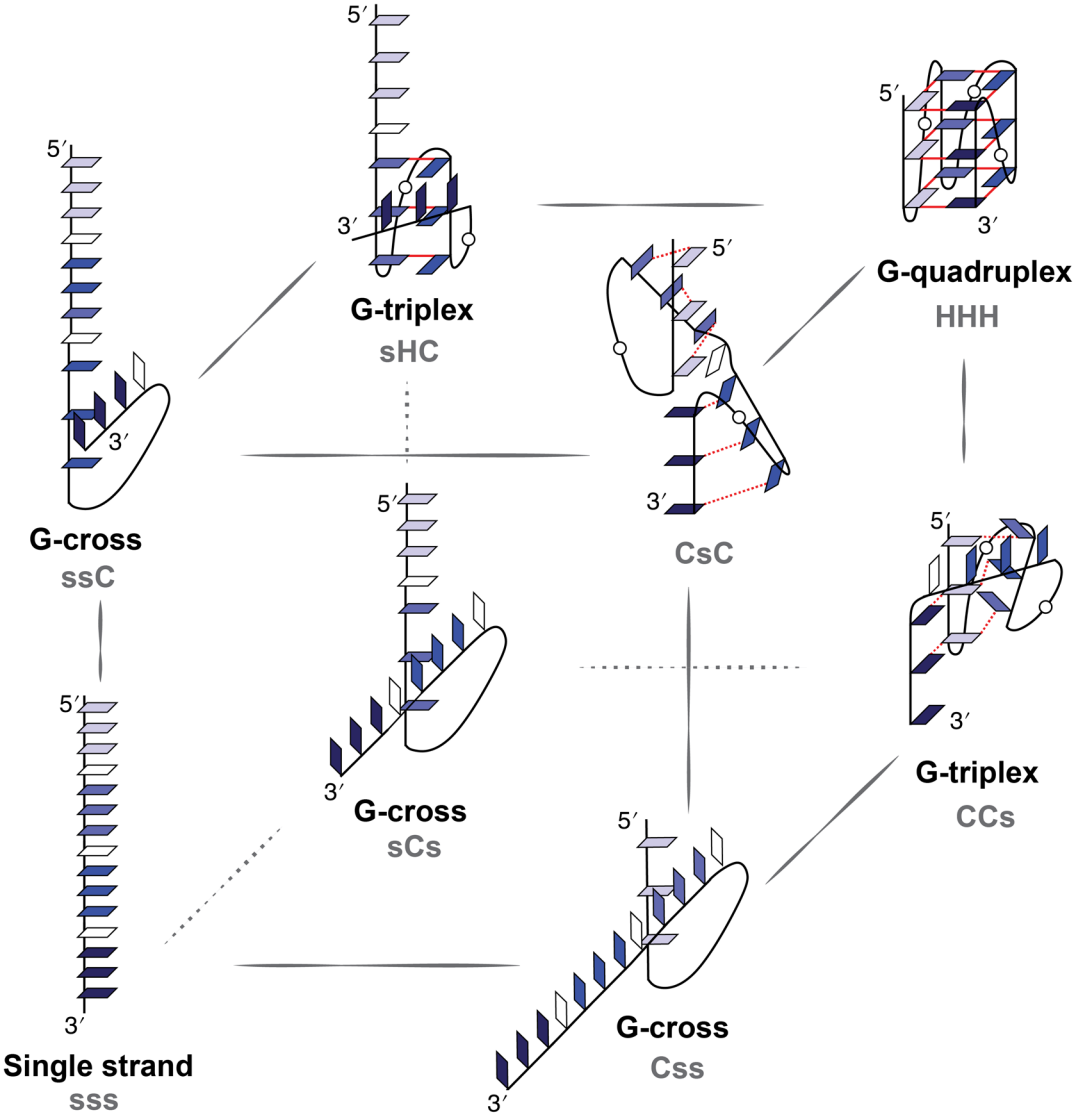
Possible folding pathway leading to the all‐anti parallel‐stranded GQ.

In the ST‐MetaD simulations, two CVs were utilized to track the position of a point in configurational space relative to a predefined path. For this purpose we adopted the concept of pathCV proposed by Branduardi et al. [104], with milestone distances calculated based on the RMSD or combination of *ε*RMSD [105] and RMSD. The first CV, referred to as pathCV, delineates the transition from one state to another through a series of milestones, while the second CV (usually referred to as *z* coordinate) measures the orthogonal distance from the pathCV. Conformations representing the minimum energy path (MEP) of the desired folding process, optimized by NEB, served as milestones. The folding pathway differed for the 7‐mer, 11‐mer, and 15‐mer. For the 7‐mer, single pathCV with 42 milestones was utilized. The folding of the 11‐mer involved a two‐step approach: first, a 2D ST‐MetaD simulation using two pathCVs, each describing the folding of either of the hairpins within the G‐triplex (Figure 1) with 42 milestones, similar to the approach used for the 7‐mer. This was followed by two ST‐MetaD simulations, each represented by one of the two NEB‐optimized MEPs, utilizing 75 milestones. Similarly, the folding of the 15‐mer was divided into 12 parts (Figure 1), each sampled using one ST‐MetaD with one pathCV consisting of 40 milestones. To ensure uniform sampling, two additional milestones were added before the first and after the last milestone. Additionally, the second variable, the Z coordinate that is the orthogonal distance to the pathCV, was restrained using harmonic restraint starting at 0.81 Å^2^ with force constant of 17.2 kcal mol^−1^ Å^−4^. All ST‐MetaD simulations employed Gaussian hills with a width of 0.4 a u for pathCV and 0.4 Å^2^ for *z* coordinate, an initial height of 0.956 kcal mol^−1^, and a deposition frequency of 50 ps. The bias factor (T + ΔT)/T was set to 35, and the deposited hills were stored on a grid. The ST‐MetaD simulations involved 10 replicas for the 7‐mer, and 16 replicas for the 11‐mer and 15‐mer, respectively. Each ST‐MetaD simulation ran for 2 μs per replica within the effective REST2 temperature range of 298–491 K for 7‐mer (the scaling factor λ values ranged from 1.0 to 0.607) and 298–509 K for 11‐mer and 15‐mer (the scaling factor λ values ranged from 1.0 to 0.586). Replica exchanges were attempted every 10 ps, and the nonbonded and dihedral parameters of the solute were scaled to achieve an average exchange rate of approximately ~ 25% between neighboring replicas.

## 3 Results

### 3.1 General Design of a Folding Pathway

In the initial simulations of hairpin loop folding we used WTMetaD combined with RMSD‐based pathCV (details are provided in the Supporting Information S1). The analysis of this simulation reveals entrapment of the GQ hairpin in various conformations stabilized by local interactions, mainly base pairing, and base stacking (Figure S6). To address this conformational entrapment, we refined the metric used in pathCV by integrating RMSD and εRMSD [105] as follows:

$$\sqrt{\frac{1}{2}\cdot {\text{RMSD}}^{2}+2\cdot C\cdot {\text{εRMSD}}^{2}}$$

where *C* is constant equaling to 1 Å^2^ to combine units of RMSD and εRMSD metrics. The prefactors 0.5 and 2 in the equation were chosen to ensure that RMSD and εRMSD values of 1 Å and 0.5 a u, respectively, being considered as cutoffs for structural similarity, contribute equally. This metric is sensitive to both the global conformational rearrangements (as captured by RMSD) as well as the local fine structural details associated with the formation and disruption of the stacking and pairing interactions that are in turn well described by εRMSD. This refined pathCV, integrating RMSD and εRMSD metrics (pathCV_RMSD/εRMSD_), enhances sampling, yet entrapment related to the sugar‐phosphate backbone substates persists, as depicted in Figure S8. Consequently, to accelerate the sampling we opted to combine the WTMetaD and REST2 into the scheme of parallel tempering metadynamics (ST‐MetaD). As shown below, the combination of ST‐MetaD with RMSD/εRMSD‐based pathCV achieved comprehensive sampling of GQ hairpin, triplex and even full GQ along a given conformational path.

**FIGURE 2:**
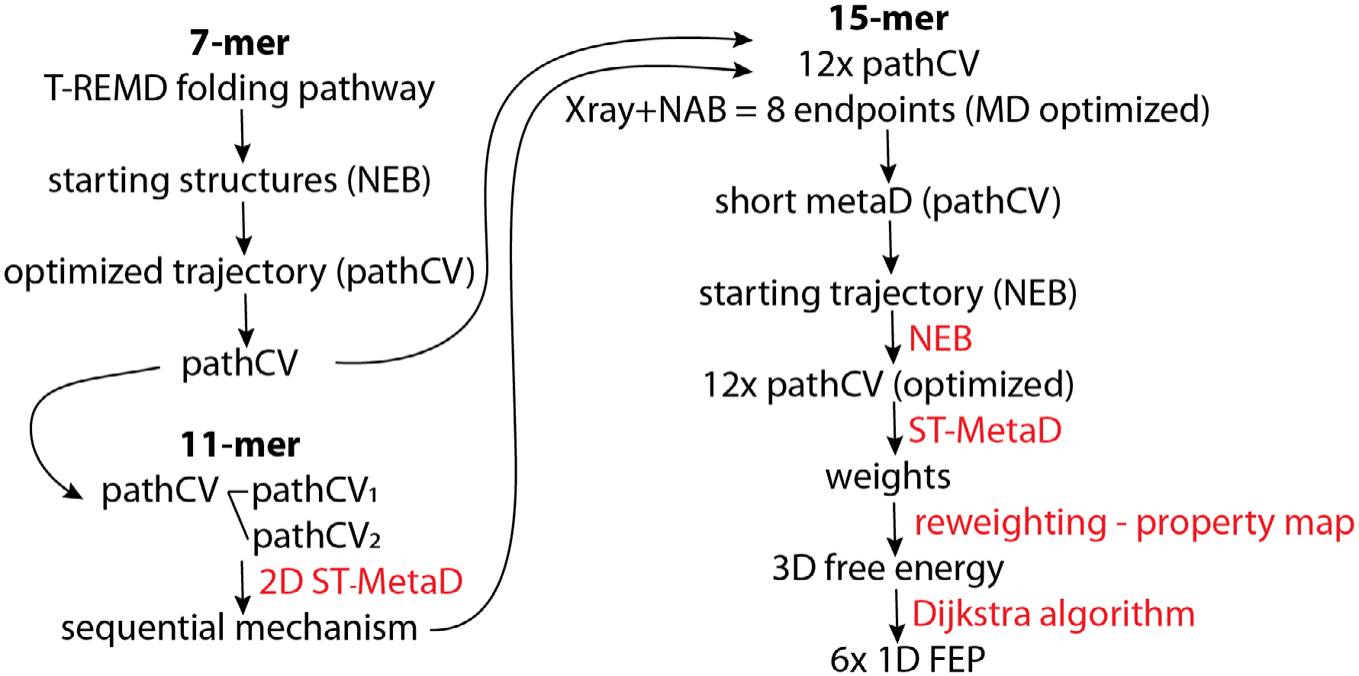
Workflow for preparing structures and pathCVs for calculation of free energy profiles of hairpin (7‐mer), triplex (11‐mer) and complete GQ (15‐mer) folding.

We focused on the all‐*anti* parallel GQ topology and developed a workflow specifically tailored for its folding (Figure 2). Utilizing insights from the identified folding pathway of the hairpin loop [57], we employed it as a guiding framework to define the pathCV variable(s) to explore the possible mechanisms of the triplex and GQ folding.

### 3.2 The Folding Pathway of the GGGAGGG Hairpin and Its Refinement Using Enhanced Sampling

The folding of G‐hairpin is a crucial step not only in various stages of GQ folding but also in the subsequent interconversion between different triplex and GQ arrangements [31, 44, 106, 107, 108, 109, 110]. Recent studies employing replica exchange simulations have suggested inherent instability of isolated parallel hairpins with propeller loops [55, 57]. These are formed transiently and are prone to a swift transition back to structurally related cross‐hairpin ensemble, which can thus be considered as a precursor to the final hairpin formation [55, 57]. Despite the low population of folded hairpin states, previous T‐REMD simulations starting from unfolded states were able to reveal the folding pathway of the d(GGGAGGG) 7‐mer. Using the insight from these simulations, we designed 40‐milestones pathCV describing G‐hairpin loop folding and optimized it using the NEB approach [77] (see Section 2 and Supporting Information S1 for details and Tables S1, S5, and S10).

**FIGURE 3:**
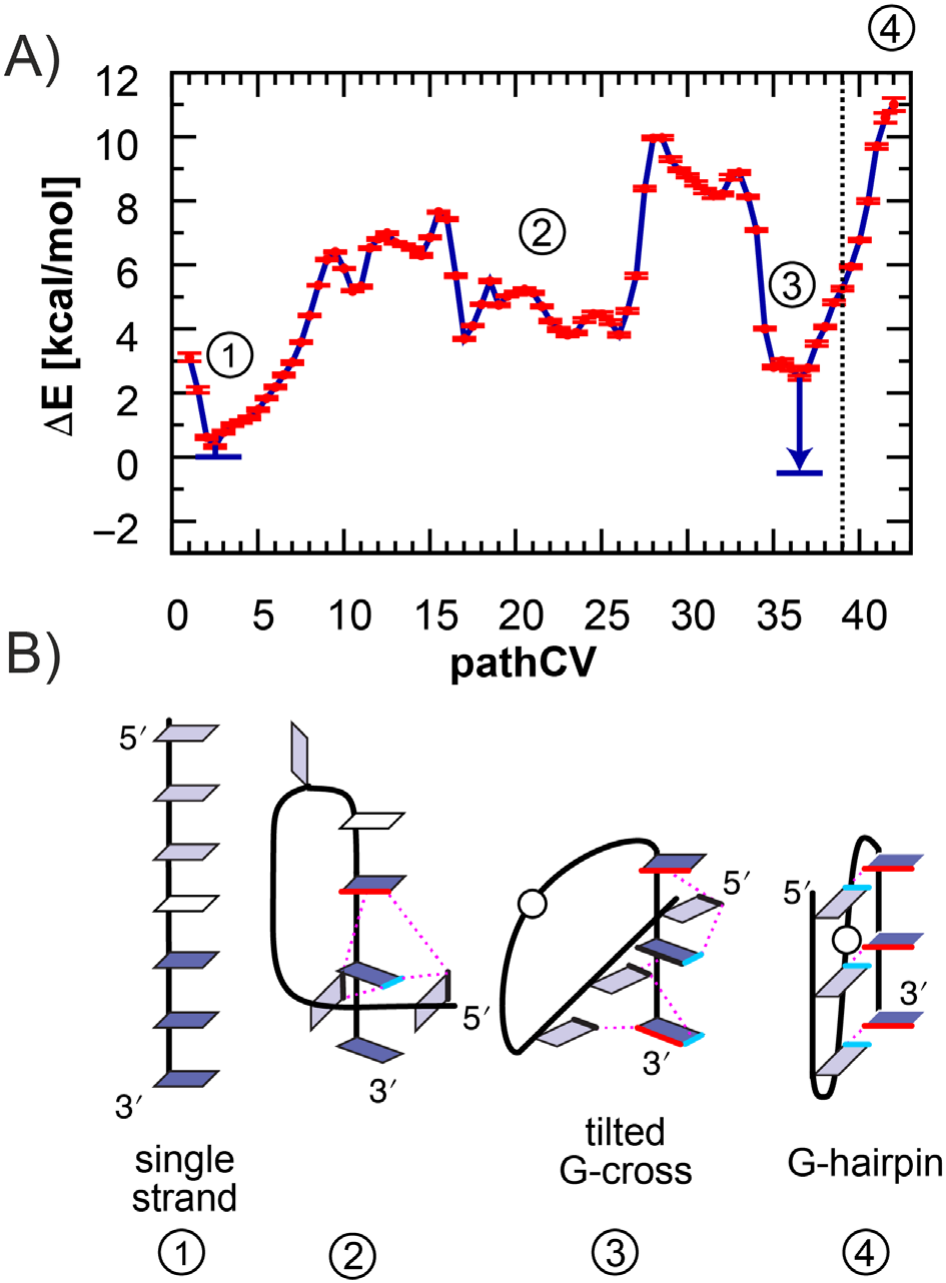
(A) Free energy profile of the parallel hairpin folding pathway, with error bars representing the standard error of the mean (SEM) indicated by red lines. The blue arrows denote free energy corrections for bias resulting from restraints on the orthogonal distance to pathCV (*z* coordinate). The dotted vertical line indicates the fully folded GQ hairpin state. (B) Representative structures along the free energy profile (guanines are shown in light and dark blue, adenines are in white, magenta lines correspond to hydrogen bonding, interacting Watson–Crick and Hoogsteen edges are highlighted in cyan and red, respectively).

The integration of ST‐MetaD and the RMSD/εRMSD metric in pathCV resulted in a transition path simulation that achieved sufficient sampling within 2 μs, capturing multiple complete transitions from the unfolded to the native state in each demultiplexed (coordinate following) replica (Figure S10). As an additional measure of convergence, we compared the free energy profile (FEP) along pathCV obtained from simulations of varying lengths. The ΔE values exhibited minimal change beyond 1.5 μs (Figure S9), as confirmed by the error bars of the FEP, which did not exceed 0.3 kcal mol^−1^ (Figure 3A). The error bars henceforth represent the standard error of the mean (SEM), see Supporting Information S1 for more details.

To ensure that the ST‐MetaD simulations sample conformations around the transition path defined by pathCV, we applied restraints to the *z* coordinate, that is, the orthogonal distance to pathCV. Specifically, structures more than 0.81 Å^2^ away from pathCV were subject to penalization by a harmonic potential (see Section 2). However, this restraint limits the sampling of distant conformational substates, such as various G‐cross conformations, thereby introducing bias into the estimation of free energy for the minima on the transition path. To address this bias, we calculated free energy corrections to all free energy minima along pathCV, corresponding to the relaxation of this restraint bias. For this purpose, we performed metaD simulation to sample the *z* coordinate (the orthogonal distance to pathCV), at given pathCV value (see SI). Conceptually, this calculation is similar to the correction usually computed in alchemical simulations of protein‐ligand binding to compensate for restraints applied to avoid ligand diffusion. The free energy corrections for single strand and G‐cross states were determined to be 0.3 ± 0.03 and 3.1 ± 0.1 kcal mol^−1^, respectively.

The free energy profile of GQ hairpin folding (Figure 3A) exhibits two major free energy minima. The first corresponds to the single‐strand structure, while the second corresponds to the G‐cross conformation. Consistently with observations from published REST2 and T‐REMD simulations, the fully folded GQ hairpin state appears to be inherently unstable in its isolated form [55, 57].

### 3.3 Triplex Folding Pathway

**FIGURE 4:**
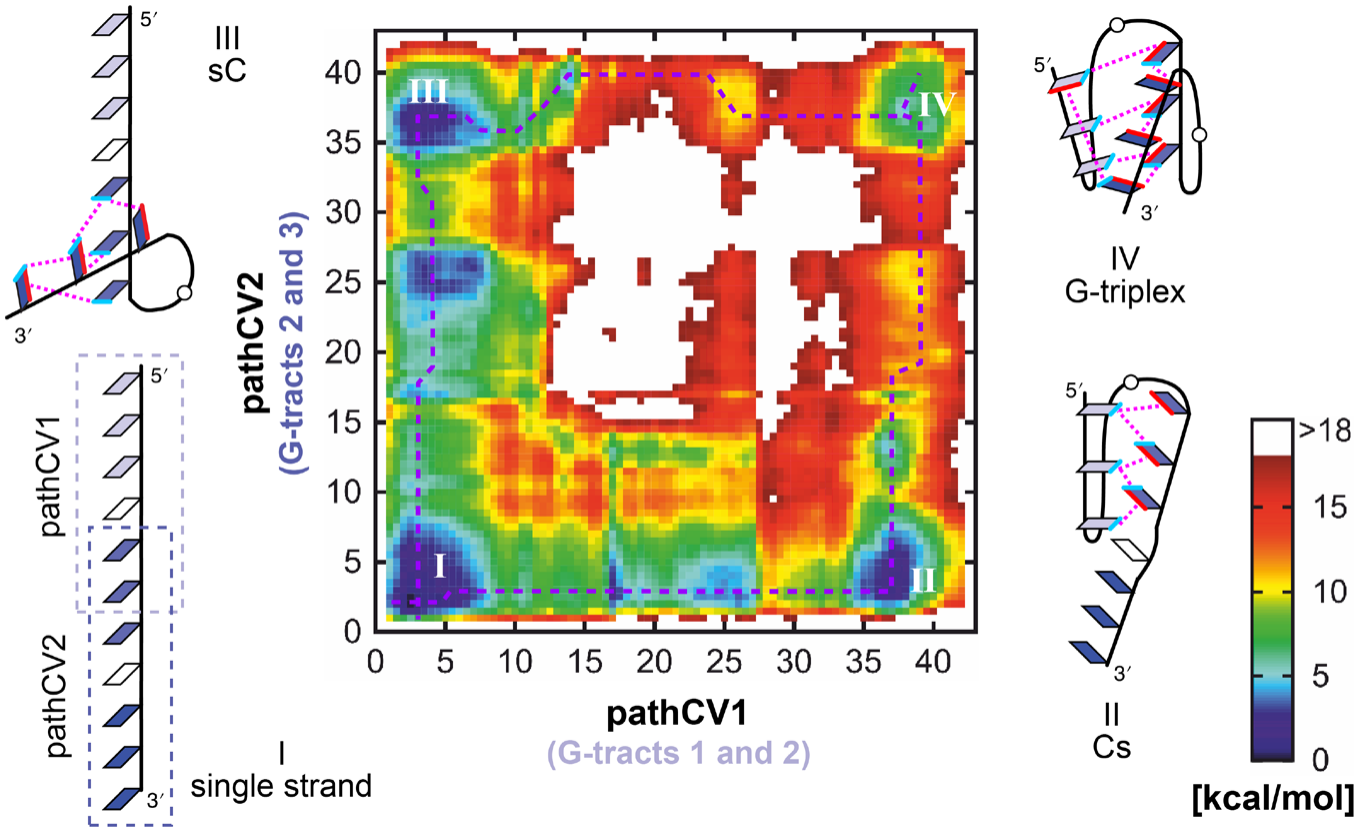
Free energy surface (FES) of the 2D ST‐MetaD simulation of 11‐mer, highlighting four main minima (I‐IV) and two minimum energy paths (dashed violet lines) from single strand to G‐triplex. For each minimum, a structural scheme of a representative structure is shown, with guanines depicted in shades of blue, hydrogen bonds indicated by magenta dotted lines, and interacting edges are highlighted in cyan and red for Watson–Crick and Hoogsteen edges, respectively.

The exploration of the d(GGGAGGG) 7‐mer GQ hairpin folding, which achieved sufficient sampling through the integration of ST‐MetaD and the RMSD/εRMSD metric in pathCV, inspired us to extend this approach to the folding of a more complex structure, namely d[(GGGA)_2_GGG] triplex (11‐mer). Initially we approached the triplex folding process with two distinct pathCVs, each outlining the folding of one of the GQ hairpin loops of the G‐triplex (Figure 4 bottom left) and combined in 2D ST‐MetaD (see Section 2 in SI). These pathCVs were constructed based on milestones derived from the 7‐mer folding transition path. PathCV1, corresponding to the folding of 5′‐G‐hairpin, consisted of milestones spanning bases G1–G6, excluding the final G7 guanine to prevent unintended interconnection between the pathCVs. PathCV2, representing the folding of 3′‐G‐hairpin, involved milestones spanning entire 3′‐G‐hairpin, that is, bases G5–G11.

The 2D ST‐MetaD simulation achieved sufficient sampling within 2 μs, capturing at least one folding‐unfolding‐refolding transition in each demultiplexed (continuous) replica, with estimated statistical errors of the 2D free energy surface (FES) below 2 kcal mol^−1^ (see Supporting Information S1, Figures S14–S16, for convergence analyses). The resulting 2D FES reveals four primary local minima: single‐strand (ss), G‐triplex like structures, G‐cross‐single‐strand (Cs) and single‐strand‐G‐cross (sC) conformations, along with two additional minima corresponding to intermediates of ss → Cs or ss → sC folding transitions. The 2D FES elucidates the sequential mechanism of G‐triplex folding, characterized by two minimum energy paths (MEPs) connecting ss and G‐triplex like structures, either through the folding of 5′‐G‐hairpin followed by 3′‐G‐hairpin, or vice versa (see Figure 4). The initial folding steps of both MEPs, ss → Cs and ss → sC, are energetically similar and overcome lower energy barriers compared to the folding of the second GQ hairpin loop, Cs → CC and sC → CC (Figure 4).

The folding barriers of the later forming loop may be overestimated in 2D ST‐metaD simulations because the folding path of the hairpins, as defined by pathCV milestones, were optimized for an isolated hairpin without considering the structural context of the second hairpin loop. As a result, the latter forming hairpin is subject to structural clashes with the already formed G‐cross. In addition, the restraints applied to the Z coordinate, i.e., the orthogonal distance to pathCV, prevent the latter forming hairpin from relaxing properly during the folding process (see Figure S13 in the Supporting Information S1 for the analysis of the clashes between CV1 and CV2 milestones). These restraints also imply that the existence of these two distinct pathways might be a consequence of the simulation setup.

To relax the observed folding paths of G‐triplex, we extracted new milestones form the 2D ST‐MetaD simulation along each of the obtained MEPs. These milestones were then optimized using NEB simulations to define new pathCVs for ss → sC → CC and ss → Cs → CC folding mechanism, respectively (see Supplementary Methods). Each pathCV consisted of 75 milestones. Subsequently, we calculated the free energy profiles along these optimized transition paths using two additional 1D ST‐MetaD simulations.

**FIGURE 5:**
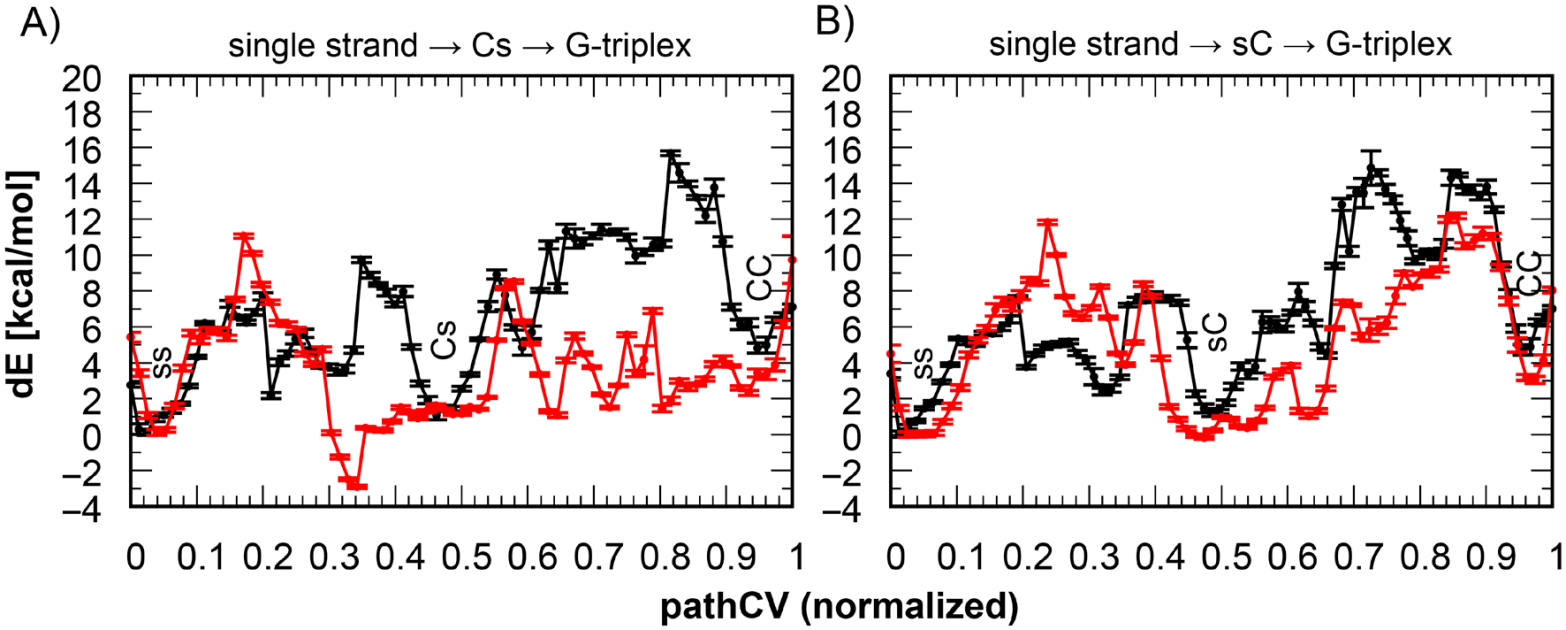
The free energy profiles (FEPs) of the folding of a parallel G‐triplex along pathway passing through G‐cross structure at (A) 5′‐side and (B) 3′‐side. The FEPs along minimum energy path (MEP) identified in the 2D ST‐MetaD (black line) is compared with the corresponding FEPs of optimized folding paths. The pathCV is normalized to facilitate comparison.

As in previous cases, both ST‐MetaD simulations show sufficient sampling after 2 μs, with statistical error below 1.3 kcal mol^−1^ (see Tables S8 and S9 and Figures S17–S20 in the Supporting Information S1). A comparison of the resulting FEPs from these ST‐MetaD simulations with those obtained from FES of the 2D ST‐MetaD simulation confirms that the Cs → CC and sC → CC transitions in the original 2D ST‐MetaD simulation followed a non‐optimal pathway (Figure 5). NEB relaxation was crucial for sampling the optimal folding paths and optimizing the interactions between G‐tract 1 and G‐tract 3 during folding (Figure S21). The FEPs indicate that ss → Cs → CC path reveals a more energetically favorable folding of the latter‐folding hairpin loop (at 5′‐side), nonetheless, the formation of the former‐folding hairpin loop is the rate limiting step in both paths (Figure 5).

### 3.4 Folding of the GQ

**FIGURE 6:**
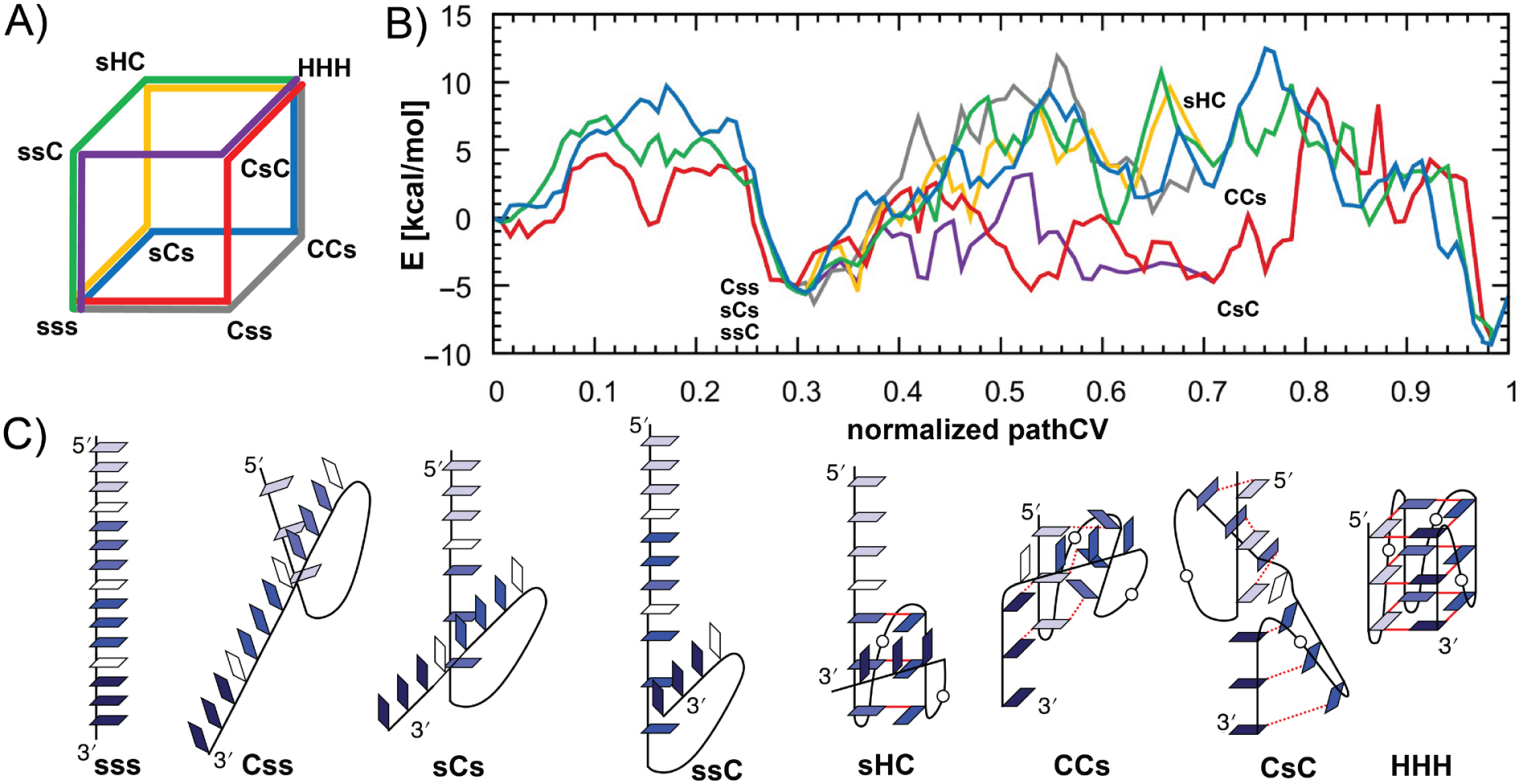
(A) The 3D cube representing six unique folding pathways from single strand to fully folded parallel GQ, each depicted in a different color. The folding states at the vertices are labeled based on the state of three consecutive G‐harpins, where “s” stands for single strand, “C” for G‐cross, and “H” for G‐hairpin. (B) The FEPs of the six pathways are shown in color scheme corresponding to paths on panel A. (C) Structural schemes of all folding structures at vertices of the cube.

Finally, we applied the abovementioned simulation protocol to map the folding pathways of the complete all‐*anti* parallel d[(GGGA)_3_GGG] GQ. The sequential folding of G‐hairpin loops results in six unique folding pathways, which can be represented by paths along the edges of a 3D cube connecting opposite vertices (Figure 6). The eight vertices of the cube correspond to different combinations of the G‐hairpin loop folding states, including the fully folded GQ and fully unfolded ssDNA. Three sets of parallel edges represent the folding of each consecutive G‐hairpin loop (Figure 6). Initially we prepared models of unfolded, folded, and partially folded states at the vertices and relaxed them using short standard MD simulations (see Section 2). These states were subsequently connected by sets of milestones based on the 7‐mer folding transition path template and then optimized by NEB method (see SI for more details on structure preparation and NEB simulations). Consequently, the six possible folding mechanisms were divided into 12 folding steps, each representing an edge of the 3D cube and described by its own pathCV. The sampling along the individual edges were then obtained using ST‐MetaD simulations based on a single pathCV. All data from these 12 individual ST‐MetaD simulations were subsequently remapped using reweighting and binless WHAM [111, 112, 113] algorithms into three‐component property map collective variable [114, 115] to obtain the 3D FES (see Supporting Information S1). Subsequently, the 3D FES was analyzed, and six MEPs representing unique folding pathways were identified by Dijkstra's algorithm [116] using an *in*‐*house* script (see SI for description of the algorithm). These pathways are presented as six FEPs of the GQ folding (Figure 6B). All 12 ST‐MetaD simulations reveal sufficient sampling after 2 μs with statistical error below 1.4 kcal mol^−1^ (Figure S24 in the Supporting Information S1). Similarly as in the case of G‐hairpin folding, we calculated free energy corrections for bias resulting from restraints on the *z* coordinate, being the orthogonal distance to pathCV (see Table S11 in the Supporting Information S1).

The obtained FEPs indicate that all six unique folding pathways are energetically comparable, suggesting that the folding of parallel GQ proceeds via a multichannel mechanism. In each pathway, the initial folding of the first G‐hairpin is separated from the folding of other two loops by well‐defined free energy minimum (Figure 6B). Among these initial steps, the initiation of folding at the 5′‐side G‐hairpin is slightly more energetically favorable, as it proceeds through a smaller energy barrier compared to the initiation at the two G‐hairpins. In subsequent steps, pathways passing through the CsC state, where both the 5′‐side and 3′‐side G‐hairpin loops are in G‐cross state, show smaller free energy barriers compared to other mechanisms. However, the free energy barriers of the folding of the third hairpin, which completes the GQ, are similar across all pathways, ranging from 10 to 13 kcal mol^−1^, and represent the highest free energy barrier, making this step rate‐determining (Figure 6B). This is likely due to the entropic barrier that needs to be crossed to align the fourth strand with the other three strands. This final formation of fully folded GQ revealed vertical strand slippage in all observed pathways.

**FIGURE 7:**
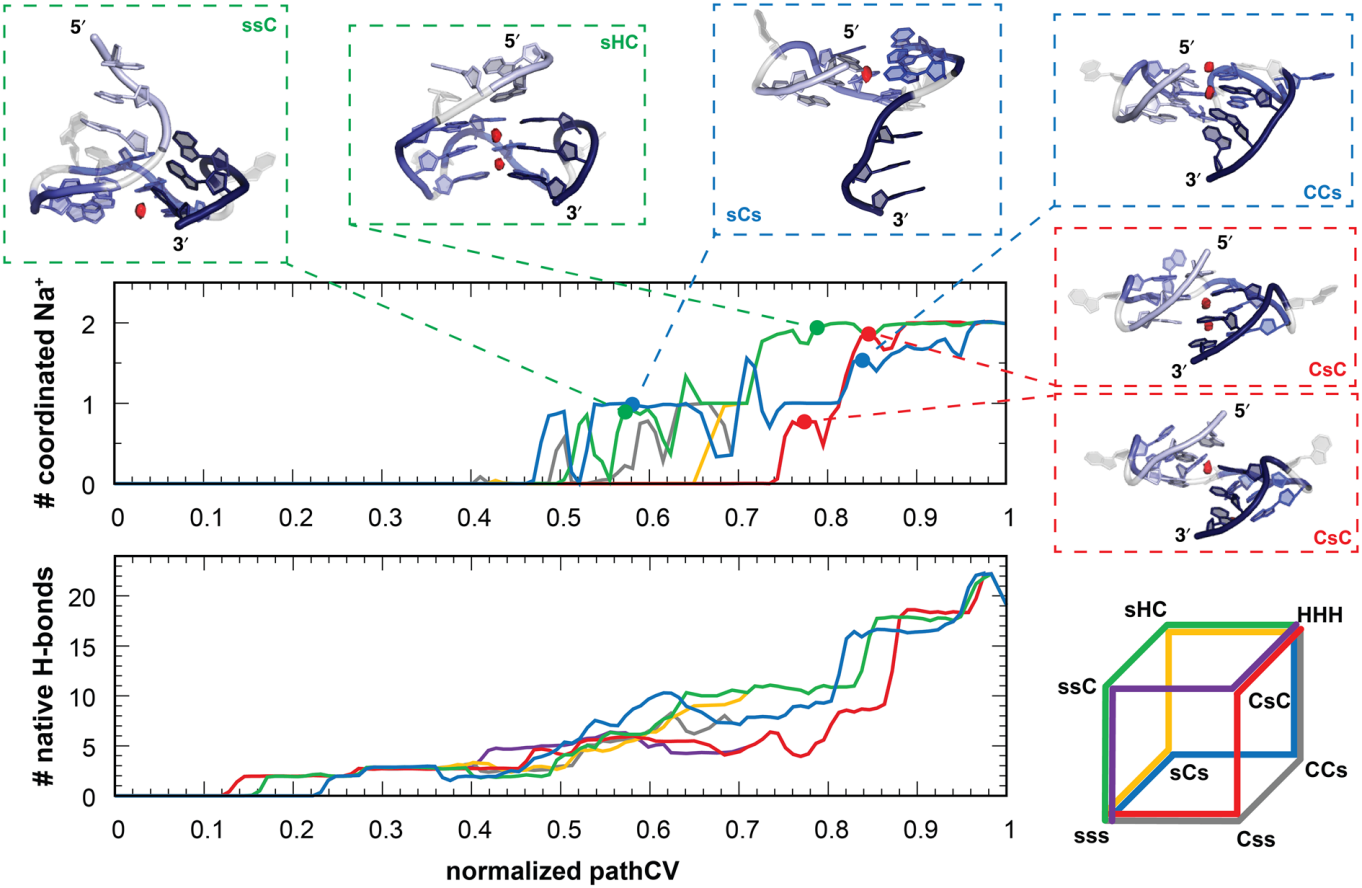
Average number of cations with at least tetracoordination to the guanines (upper graph) and the average number of native hydrogen bonds (lower graph) along the GQ folding pathway. Representative 3D structures along the folding pathways with ion binding sites highlighted by spots of high ion density are shown.

The first folding barrier of the G‐hairpin formation is associated with the loss of stacking interactions upon initial loop bending, which is later energetically compensated by formation of hydrogen bonds within guanine‐guanine interactions. As two adjacent G‐hairpin loops adopt either hairpin or G‐cross conformation (CCs or sHC states), the first cation binds to the partially formed central channel (see Figure 7). During the final stage of folding, the emerging GQ binds both cations into its central channel, typically upon the folding of the last G‐hairpin and the formation of fully folded GQ. In all pathways, the central ion channel is finally fully occupied by both cations even before the free energy drops down to the GQ free energy minimum (Figures 6 and 7). The final stabilization of the GQ fold is thus attributed to the formation of native hydrogen bond and stacking interactions, while ion binding in the central channel remains crucial for compensating the negative charges of guanine's Hoogsteen edges (located at N7 and O6 atoms) during the final folding stage.

The overall folding free energy is found to be − 9 kcal mol^−1^. As the native GQ fold is supported by 24 H—bonds not present in the single‐stranded state, this means that without the gHBfix support (increasing stability of each H—bond by 0.5 kcal mol^−1^, see Section 2), the free‐energy difference would be a substantial + 3 kcal mol^−1^, which sharply contrasts with the known stability of the GQ. Positive folding free energy of this quadruplex was calculated also by Pokorna et al. [45] (+ 17 kcal mol^−1^), which indicates a severe imbalance in the non‐modified force field without gHBfix term.

## 4 Concluding Remarks

The effort to uncover the GQ folding pathway becomes even more significant when conventional experimental and theoretical techniques do not provide a comprehensive atomistic view of the entire folding process. By developing a new protocol for the folding of the entire GQ, we have harnessed the power of computer modeling to explore the folding process at a level of detail often inaccessible experimentally. Our approach, which combines pathCV and property map methods with the NEB method and ST‐MetaD, effectively samples the complex multichannel folding pathways of complex nucleic acid system such as parallel GQ. Although state‐of‐the‐art simulation approaches such as pathCV or property map are designed to sample complex conformational transitions, their application to intricate systems like nucleic acid folding can be challenging. Our study demonstrates that combining pathCV with a combined RMSD/εRMSD metric accounting for both global and local structural changes, along with NEB and ST‐MetaD, can effectively sample very complex multichannel folding pathways of parallel DNA GQ.

We found that the folding mechanism of the all‐*anti* parallel DNA GQ proceeds via a multichannel mechanism, with the sequential folding of G‐hairpin loops. All six pathways, differing in the order of particular G‐hairpin folding, are energetically comparable. This complex mechanism thus reveal some similarities to the protein folding mechanisms that proceeds through the molten globule state, as the DNA GQ sequence can form multiple compact globular conformations, from which the folded GQ is accessible via vertical strand slippage mechanism. The strand slippage was observed to occur during the final folding stages, specifically during the folding of the last G‐hairpin, contributing to the formation of native interactions. The resulting folding barrier of 10–13 kcal mol^−1^ corresponds to this particular mechanism. However, the overall kinetics should also consider entropic traps of various *syn*/*anti* orientations and folding into numerous other different misfolded topologies and intermediates. Despite all these kinetic traps inherent to the kinetic partitioning mechanism of folding, our observations strongly support that the parallel GQ topology is structurally accessible via a multichannel mechanism involving sequential G‐hairpin loop formations directly from single‐stranded DNA.

## Author Contributions

The manuscript was written through contributions of all authors. All authors have given approval to the final version of the manuscript.

## Conflicts of Interest

M.O. has a share in InSiliBio biosimulation company.

## Supporting information


Supporting Information S1.

## Data Availability

The data that support the findings of this study are openly available in Zenodo at https://zenodo.org/records/12819538, reference number https://doi.org/10.5281/zenodo.12819538.
