# Supplementary material for: Computer Folding of Parallel DNA G‐Quadruplex: Hitchhiker's Guide to the Conformational Space
Source: J Comput Chem. 2024 Dec 9;46(1):e27535. doi: 10.1002/jcc.27535 (PMC11628365; doi:10.1002/jcc.27535)
Supplement: Supplementary file 1 — Supporting Information S1. [file JCC-46-0-s001.pdf]

Supporting Information: Computer folding of parallel DNA G-quadruplex: Hitchhiker's guide to the conformational space

Michal Janeček, Petra Kührová, Vojtěch Mlýnský, Petr Stadlbauer, Michal Otyepka, Giovanni Bussi, Jiří Šponer and Pavel Banáš

Table of Contents

Supplementary Methods ***2***
MD setup 2
Classical MD simulations 2
WT-MetaD simulations setup 2
ST-MetaD performed on orthogonal distance from the pathCV 3
Calculation of free energy surface and statistical error 3
Property map 3
Dijkstra's algorithm 4
Calculation of number of coordinated ions and number of native H-bonds 4
The 7-mer ***5***
Classical MD simulations **5**
NEB simulations **6**
WT-MetaD simulations **9**
WT-MetaD simulations of 7-mer based on RMSD pathCV 9
WT-MetaD simulation of 7-mer based on εRMSD/RMSD pathCV 12
ST-MetaD simulations **16**
ST-MetaD (s→H) of the 7-mer 16
ST-MetaD simulations of 7-mer performed on orthogonal distance to transition path 21
The 11-mer ***22***
2D ST-MetaD (ss→HH) **22**
NEB simulations for relaxation of MEPs after 2D ST-MetaD **28**
Preparation of starting structures 28
ST-MetaD ss→Cs→CC **29**
ST-MetaD ss→sC→CC **32**
The 15-mer ***35***
Classical MD simulations **37**
NEB simulations **39**
Preparation of starting structures 39
ST-MetaD simulations of 15-mer **41**
References ***45***

Supplementary Methods

MD setup

Simulations were carried out using AMBER 18[1] for system equilibration and NEB simulations, and GROMACS 2018[2-3] for ST-MetaD simulations. All simulations were performed in NVT ensemble under periodic boundary conditions at temperature of 298 K. Electrostatic interactions were treated using the particle-mesh Ewald (PME) method, with a 10.0 Å cut-off applied to non-bonded interactions. The SHAKE algorithm with a tolerance of 10^{-5} Å (AMBER) or the LINCS algorithm (GROMACS) was employed to constrain the positions of all hydrogen atoms. Additionally, hydrogen mass repartitioning[4] was used to allow integration time step of 4 fs.

Classical MD simulations

Before initiating classical MD simulations, the solvated starting structure underwent optimization and heating using a well-established simulation protocol. Initially, the solute hydrogens were minimized, followed by the minimization of solvent including ions, utilizing the steepest descent method. Subsequently, the solvent was heated up to 298 K during 500-ps MD run under NpT conditions (pressure=1.0 atm) to facilitate solvent mixing and density relaxation. Then, a series of minimizations of the DNA molecule was performed with positional restraint applied to the sugar-phosphate backbone, gradually decreasing the force constant from 1000 kcal/mol to zero. Finally, the entire simulation box was heated to 298 K during 100-ps long MD run under NVT, followed by another 100-ps long MD under NpT (pressure=1.0 atm; temperature=298 K) to relax the density. Weak-coupling thermostat[5] was applied under NVT conditions, while the same thermostat along with Berendsen barostat[5] were used under NpT conditions. In these initial MD simulations, a 2 fs integration timestep was employed along with SHAKE[6] algorithm to fix the positions of hydrogens with respect to the rest of the structure.

Classical MD simulations were conducted under NVT conditions at 298 K with a weak-coupling thermostat. The SHAKE algorithm, combined with the hydrogen mass repartition[7] approach, facilitated a 4 fs integration timestep.

WT-MetaD simulations setup

Two initial attempts were made to sample the conformational space of the 7-mer using only WT-MetaD. These simulations utilized parameters similar to those described for ST-MetaD in the main text (see Methods section in the main text). In the first WT-MetaD simulation, the pathCV was defined based solely on RMSD, resulting in equidistant milestones in RMSD selected from the NEB simulations (Selection 1, Table S2). During this simulation, the orthogonal distance from the pathCV was restrained by a harmonic potential starting at 0.81 Å^2 with a force constant of 17.2 kcal·mol^{-1}·Å^{-4}. Thus, the transition path, defined by milestones, was surrounded by a cylindrical area that was not biased, which extended up to εRMSD and RMSD values of 0.57 a.u. and 1.15 Å, respectively. The second WT-MetaD simulation employed the same parameters as the ST-MetaD described in the main text, including the pathCV definition based on a combination of εRMSD and RMSD, with milestones selected accordingly (Selection 2, Table S3). Selection 2 aimed to achieve equidistant milestones based on a combined εRMSD+RMSD metric.

ST-MetaD performed on orthogonal distance from the pathCV

In these ST-MetaD simulations, the MetaD bias accelerated sampling along orthogonal distance from the pathCV, so called Z coordinate, with fixed value of pathCV and significantly increased cylindrical area around pathCV that was not biased. Gaussian hills were utilized with width of 0.4 Å^2, an initial height of 0.956 kcal/mol, and a deposition frequency of 100 ps. The bias factor (T + ΔT)/T was set to 35, and the deposited hills were stored on a grid with bins spaced every 0.08 Å^2. The Z coordinate was sampled up to the value of 4.0 Å^2 from pathCV, which corresponds to the cylindric that was extended up to εRMSD and RMSD values of 2.83 a.u. and 5.66 Å, respectively. After this boundary we applied a harmonic restraint with a force constant of 11.0 kcal.mol^{-1}.Å^{-4}. Simultaneously, the pathCV was restrained with a force constant of 2.75 kcal.mol^{-1}.a.u.$^{-2}$ to keep the system at the beginning or end of the pathway, below or above the given milestone of the pathCV, as summarized in Table SX in "Milestones" column. The number of replicas in these ST-MetaD simulations, along with the effective REST2 temperature range, remained the same as in the previous ST-MetaD performed on the pathCV, with 10 for the 7-mer and 16 replicas for 15-mer, respectively.

Calculation of free energy surface and statistical error

Reweighting and WHAM

The final Free Energy Surfaces (FES) were calculated using the sampling data obtained from the ST-MetaD simulations, employing the weighted histogram analysis method (WHAM)[75] and reweighting with final bias potential[76]. Reweighting was performed utilizing trajectories from all replicas of the ST-MetaD simulations. The energy bias calculated for each snapshot *i* and each replica with scaling factor λ using the formula:

$$V_{i,\lambda} = \Delta E_{\lambda \to \lambda_0}(\boldsymbol{X}_i) + V_{MetaD,\lambda}(\boldsymbol{X}_i),$$

where $\Delta E_{\lambda \to \lambda_0}(\boldsymbol{X}_i)$ represents the difference between potential energy of the simulated system with coordinates $\boldsymbol{X}_i$ at given Hamiltonian scaled by factor λ and the potential energy of the same coordinates calculated at the reference unscaled Hamiltonian with $\lambda_0 = 1$. $V_{MetaD,\lambda}(\boldsymbol{X}_i)$ represents the MetaD-bias energy at replica with Hamiltonian scaled by factor λ calculated from the final bias potential.

The weighting factors of every snapshot in all replicas were subsequently determined using the Weighted Histogram Analysis Method (WHAM). From the obtained weights we finally calcualted the histogram along given collective variable(s), which was subsequently transformed to the FEP or FES. The same protocol was used also for remapping the twelve ST-MetaD simulations onto CV coordinates of the single property map.

Statistical errors

Statistical errors associated to data points on FEP or FES were expressed as standard errors of the mean (SEM) and assessed through block-average analysis.[8] The snapshots were sorted in demuxed coordinate-following replica and each replica represented a single block, so that the statistical error finally represented variability between coordinate-following replicas. The replicas that failed to sample complete CV space (i.e., some bins of the FES histogram were populated by less than 200 frames) were excluded from the analysis.

Property map

The 12 ST-MetaD simulations, corresponding to the twelve edges of the cube, were aggregated together using remapping the simulations into one three-dimensional property map collective variable.[9-10] The milestones were associated with three-dimensional property formed by x, y, z

coordinates corresponding to the position of the milestone on the cube. As the pathCVs in each ST-MetaD simulation were defined by 40 milestones, the property map x, y, z coordinates ranges in the indexes from 1 to 40, so that the fully unfolded state sss corresponded to value [1, 1, 1], while the fully folded HHH state was defined by milestone [40, 40, 40]. Using this property map CV, we were able to assign x, y, z coordinate to all snapshots from all 12 ST-MetaD simulations. We thus aggregated data from these simulations and calculated FES of the complete scheme of folding pathways as a function of x, y, z property map coordinates using the above mentioned reweighting and WHAM algorithm. This 3D FES was subsequently analyzed by Dijkstra's algorithm.

Dijkstra's algorithm

Dijkstra's algorithm was employed to identify the minimum energy pathways (MEPs) connecting selected nodes within a 3D FES obtained by remapping to the property map coordinates. Following the remapping of the 12 ST-MetaD simulations into a unified 3D property map, the system explored the expanded 3D conformational space, encompassing not only the edges of the cube but also part of its interior around the edges and vertices. Consequently, we utilized Dijkstra's algorithm to ascertain the MEPs between the single-stranded and G-quadruplex states across the sampled 3D space. As expected, the each of the obtained MEP was located near three cube edges connecting opposite vertexes associated with unfolded sss and folded HHH states. In order to find all possible MEPs, we blocked the obtained MEP by introducing high energies around the center of the middle edge and apply the Dijsktra's algorithm again. In total, six different MEPs corresponding to six different combination of cube edges connecting the vertexes associated with unfolded sss and folded HHH states were identified in the property map space. Subsequently, we extracted the free energy along these MEPs into a single FEP (see Figure 6B in the main text).

Calculation of number of coordinated ions and number of native H-bonds

To determine the number of Na+ cations coordinated by the 15-mer, we counted the number of Na+ cations with at least four G(O6) atoms within distance below 3.0 Å for every frame of reference replica. Subsequently we calculated the average number of coordinated Na+ cations along the folding pathway.

The criteria for counting the native hydrogen bonds were heavy atom distance of bellow 3.5 Å and an X…H-Y angle above 130°. Subsequently we calculated the average number of formed native hydrogen bonds along the folding pathway.

The 7-mer

Structural dynamics of GGGaGGG 7-mer was described by nine simulations, three classical MD simulations, one NEB simulation, two metadynamics, and three ST-MetaD, see Table S1.

Table S1: Summary of all performed simulations for the 7-mer.

Conformation[a]	Method	Length [μs]	Replicas	Milestones	Starting structures
s	MD	0.5	-	-	single strand (NAB)
H	MD	0.5	-	-	G-hairpin (PDB ID:2LEE)
H	MD	0.01[b]	-	-	G-hairpin (PDB ID:2LEE)
s→H	NEB	0.49	40	-	ref. 11
s→H	MetaD[c]	3	-	42	NEB s→H (selection 1)
s→H	MetaD[d]	3	-	42	NEB s→H (selection 2)
s→H	ST-MetaD[d]	2	10	42	NEB s→H (selection 2)
s	ST-MetaD[e]	0.5	10	#1-#7	NEB s→H (selection2)
C	ST-MetaD[e]	0.5	10	#34-#42	NEB s→H (selection2)

[a] Abbreviations of conformations: s (single strand), H (hairpin), C (cross structure), s→H (path from single strand to hairpin

[b] The short 0.01-μs MD was performed with positional restraint on C2, C4, C6, C1′, C4′ and P atoms of DNA in order to keep the native conformation of G-hairpin, while the other simulation was run without this restraint.

[c] Simulations based on RMSD pathCV

[d] Simulations based on RMSD/εRMSD pathCV

[e] ST-MetaD performed only on orthogonal distance from the pathCV

Classical MD simulations

To obtain the initial structures for NEB simulations encompassing both unfolded and folded states of the hairpin, we conducted three classical MD simulations of the 7-mer (see Table S1). The first simulation focused on the single-stranded conformation (referred to as $MD_{single\ strand}$). During the initial 400 ns of this MD run, the unfolded structure remained largely unchanged from the starting conformation, as depicted in Figure S1, with occasional reversible unstacking of the terminal guanines. Subsequently, we conducted two simulations of the hairpin: one without any restraints and one with positional restraints. The hairpin simulation without positional restraints quickly unfolded toward a G-cross structure (εRMSD with respect to the G-hairpin >0.7, $MD_{hairpin\ (unrestrained)}$ in Figure S1). Therefore, to obtain the folded G-hairpin with a relaxed solvent, which was to be used as the endpoint for NEB, we conducted an additional MD simulation with positional restraints applied to the G-hairpin, specifically on the C2, C4, C6, C1′, C4′, and P atoms ($MD_{hairpin\ (restrained)}$ in Figure S1).

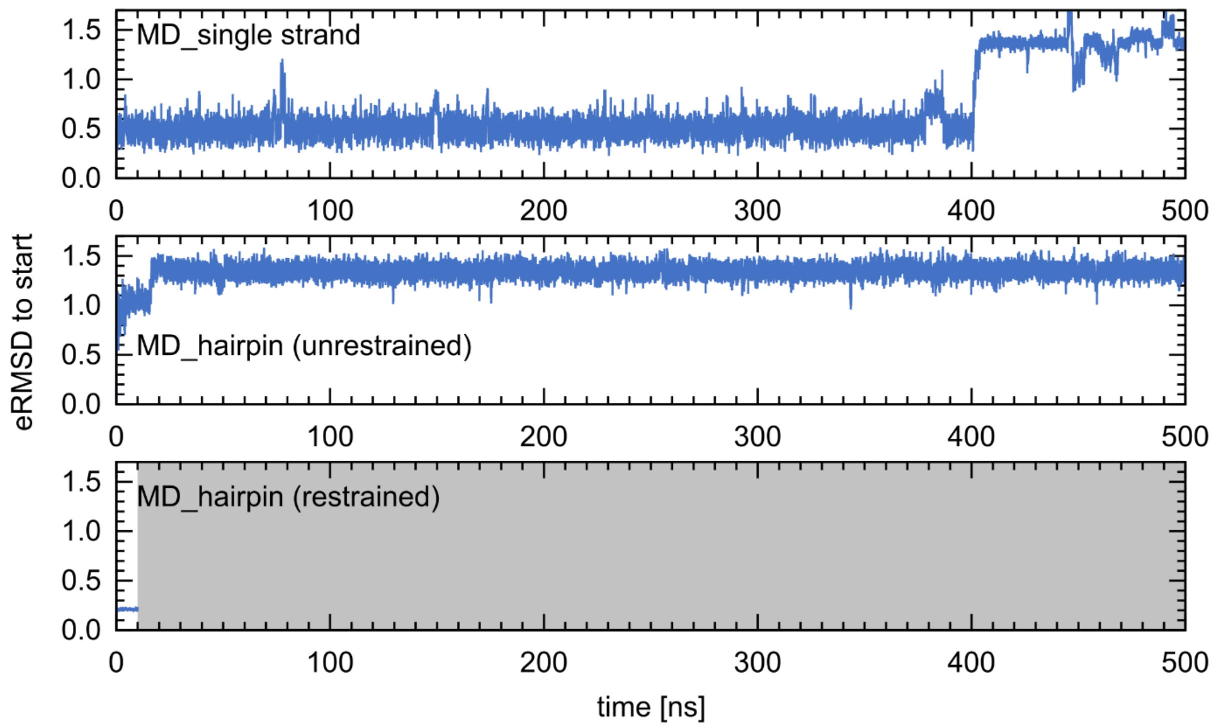

Figure S1: Time evolution of the εRMSD of the 7-mer, calculated relative to the starting structure, across three classical MD simulations.

NEB simulations

Preparation of starting structures

Milestones for the NEB (s→H) simulations were prepared using two approaches. The endpoint structures were chosen as equilibrated structures of the single-stranded and G-hairpin conformations from classical MD simulations, specifically from the MD of the single strand and MD of the G-hairpin with positional restraints (refer to Table S1 and chapter Classical MD simulations for MD details). They were then complemented by 38 milestones describing the folding pathway of the 7-mer from previous published REMD simulations.[11]

Selection of milestones for pathCV from NEB simulation

Milestones for WT-MetaD and ST-MetaD simulations of the 7-mer defining pathCV were selected in two ways:

- Selection 1 – The milestones were chosen from the final 50 ns of each NEB replica, ensuring they were evenly spaced along the transition path. This selection method aimed to minimize the RMSD variation between neighboring snapshots (see Table S2).
- Selection 2 – Similar to Selection 1, but instead of RMSD we used a combination of RMSD and εRMSD (see Table S3).

Table S2: RMSD calculated between neighboring milestones selected from the NEB simulation corresponding to Selection 1.

Number of milestone	RMSD to previous milestone [Å]
2	0.603
3	0.645
4	0.666
5	0.739
6	0.718
7	0.677
8	0.649
9	0.732
10	0.627
11	0.643
12	0.695
13	0.649
14	0.634
15	0.730
16	0.701
17	0.678
18	0.644
19	0.645
20	0.613
21	0.659
22	0.647
23	0.672
24	0.673
25	0.653
26	0.666
27	0.667
28	0.610
29	0.698
30	0.641
31	0.617
32	0.660
33	0.680
34	0.671
35	0.669
36	0.684
37	0.640
38	0.620
39	0.616
40	0.644

Table S3: RMSD, εRMSD and their combination $\sqrt{\frac{1}{2} \cdot RMSD^2 + 2 \cdot C \cdot \varepsilon RMSD^2}$ (see the main text) calculated between neighboring milestones selected from the NEB simulation corresponding to Selection 2.

Number of milestone	RMSD to previous milestone [Å]	εRMSD to previous milestone	$\sqrt{\frac{1}{2} \cdot RMSD^2 + 2 \cdot C \cdot \varepsilon RMSD^2}$ [Å]
2	0.602	0.214	0.522
3	0.751	0.225	0.619
4	0.685	0.233	0.586
5	0.703	0.216	0.584
6	0.626	0.212	0.534
7	0.667	0.261	0.599
8	0.718	0.279	0.643
9	0.704	0.392	0.745
10	0.649	0.235	0.567
11	0.796	0.439	0.838
12	0.669	0.421	0.761
13	0.605	0.307	0.609
14	0.600	0.426	0.737
15	0.593	0.412	0.718
16	0.556	0.379	0.664
17	0.720	0.449	0.814
18	0.713	0.421	0.780
19	0.671	0.312	0.648
20	0.731	0.315	0.682
21	0.738	0.271	0.648
22	0.723	0.308	0.671
23	0.689	0.244	0.597
24	0.630	0.202	0.529
25	0.738	0.349	0.718
26	0.812	0.397	0.803
27	0.610	0.420	0.734
28	0.531	0.271	0.537
29	0.640	0.313	0.633
30	0.666	0.238	0.579
31	0.714	0.216	0.590
32	0.695	0.377	0.725
33	0.625	0.373	0.688
34	0.580	0.318	0.608
35	0.746	0.304	0.681
36	0.655	0.346	0.674
37	0.756	0.387	0.766
38	0.688	0.349	0.693
39	0.630	0.394	0.714
40	0.555	0.246	0.524

Convergence of NEB simulations

Convergence of the NEB simulations was assessed by calculation of the orthogonal distance from the pathCV based on RMSD values. The final milestones from the corresponding NEB simulation served as reference structures for this calculation. Each frame of each replica was compared with these final milestones, and both the average and maximum of the orthogonal distance from the pathCV were calculated, along with their square roots. In the case of the 7-mer, no significant changes in these values were observed after 160 ns (see Figure S4). Hence, further NEB simulations were performed at 200 ns.

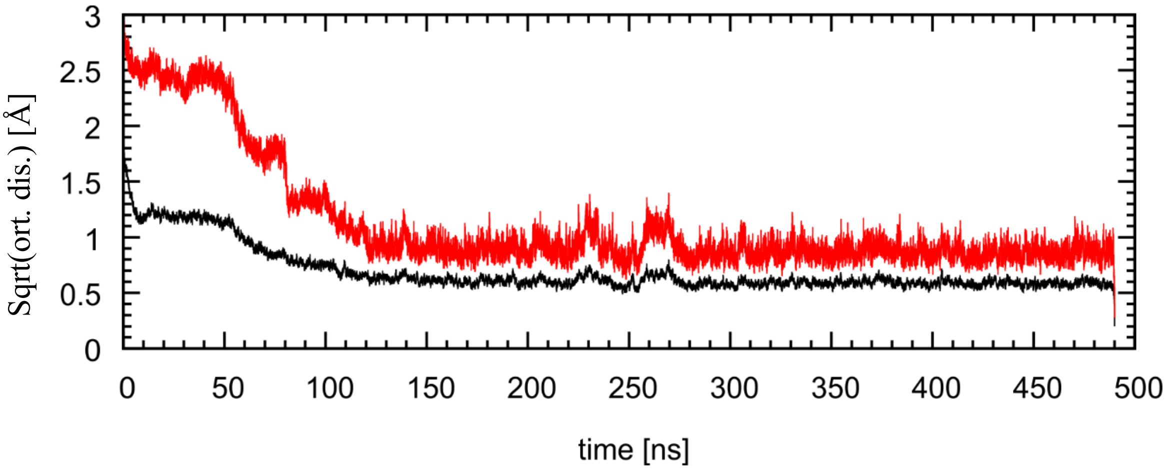

Figure S4: Convergence of the NEB (s→H) simulation of the 7-mer was assessed using orthogonal distance to transition path. The black and red curves depict the average and maximum square roots of orthogonal distance to transition path, respectively, across the 38 running replicas, respectively.

WT-MetaD simulations

WT-MetaD simulations of 7-mer based on RMSD pathCV

As a first attempt to simulate the folding of the 7-mer, we conducted a WT-MetaD (s→H) simulation with a pathCV based on the RMSD metric between milestones. However, even after 3 µs, convergence was not achieved (Figure S5). The system became trapped in certain conformations multiple times likely due to the presence of hidden variables related to base stacking interactions, H-bond patterns, and backbone conformation (see Figure S6). One notable obstacle in convergence likely acting as hidden variable corresponds to the base stacking interaction between G2 and G3 bases. This interaction is present in milestone #8 but is broken in milestone #9 (Figure S6b). Despite the MetaD bias being applied to all atoms of the 7-mer via the RMSD pathCV, it fails to accelerate the unstacking of the G2|G3 interaction, leading to the system remaining stuck at pathCV < 8.5 on the nanosecond timescale (Figure S6a). The system only proceeds to sample states at pathCV > 8.5 if the G2|G3 stacking breaks spontaneously. Similar issues are observed for the transition between milestones #33 and #34, again due to the presence or absence of the G2|G3 stacking interaction (Figure S6c). Another type of potential hidden variable arises from differences in the H-bond pattern of G2, G3, and G5 bases between milestones #12 and #14 (Figure S6d). This discrepancy causes the system to remain stuck at either of these states on the nanosecond timescale. Additionally, hidden variables involving backbone dihedral angles were observed between milestones #20 and #21 (Figure S6e). Fluctuations in the zeta dihedral angle of G3 and the alpha dihedral angle of A4 influence the system's preference for milestones at pathCV < 20.5 or > 20.5. Despite

encountering these challenges, the system was able to complete the transition from the unfolded state to the native state and back six times (Figure S6a).

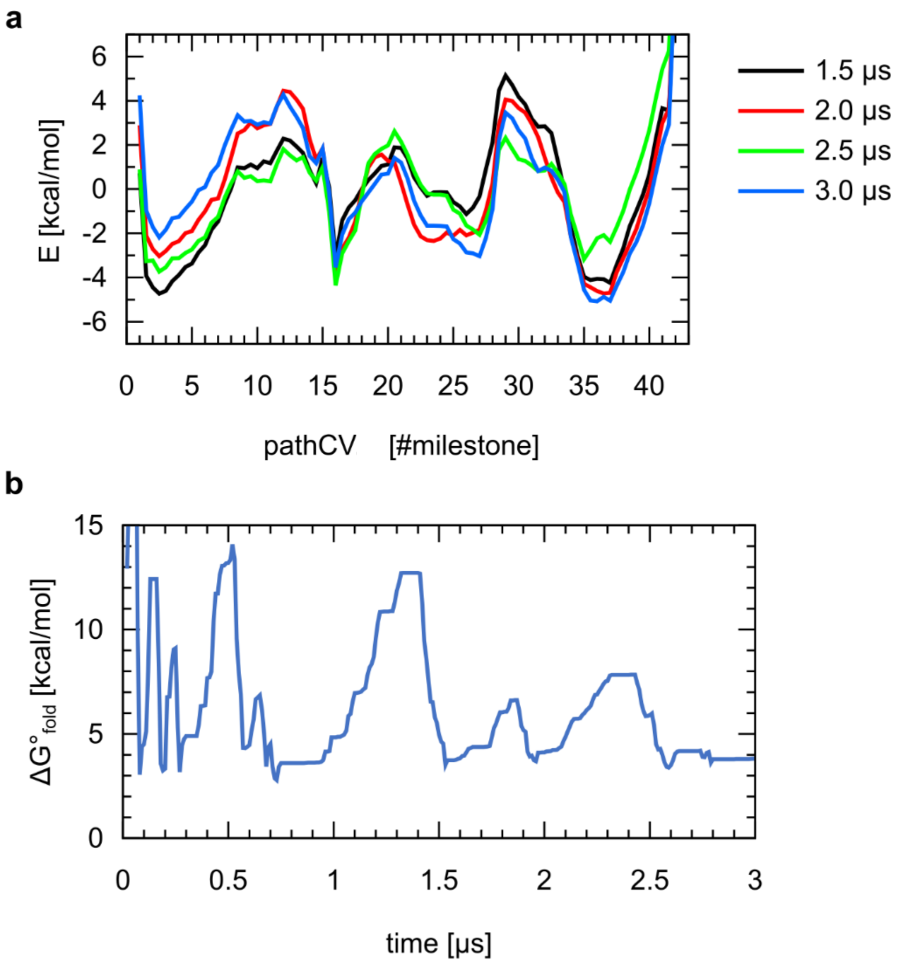

Figure S5 – Convergence of the WT-MetaD (s→H) of the 7-mer with pathCV based on RMSD. a) FEP calculated at different time intervals b) Development of the folding energy of G-hairpin ΔG°_{fold} over time. The ΔG°_{fold} was calculated using the formula: $\Delta G^{\circ}_{fold} = -RT[\ln(\Sigma_{i,native}\, w_i) - \ln(\Sigma_{i,other}\, w_i)]$, where w_i is the weighting factor of the i-th snapshot considering its current time-dependent MetaD bias. It's important to note that during the simulation, the conformational space of the 7-mer was restrained to states where the distance from pathCV was ≤0.81. Therefore, the value of ΔG°_{fold} cannot be directly compared to that of an unrestrained system and values serves only to demonstrate the convergence of the WT-MetaD.

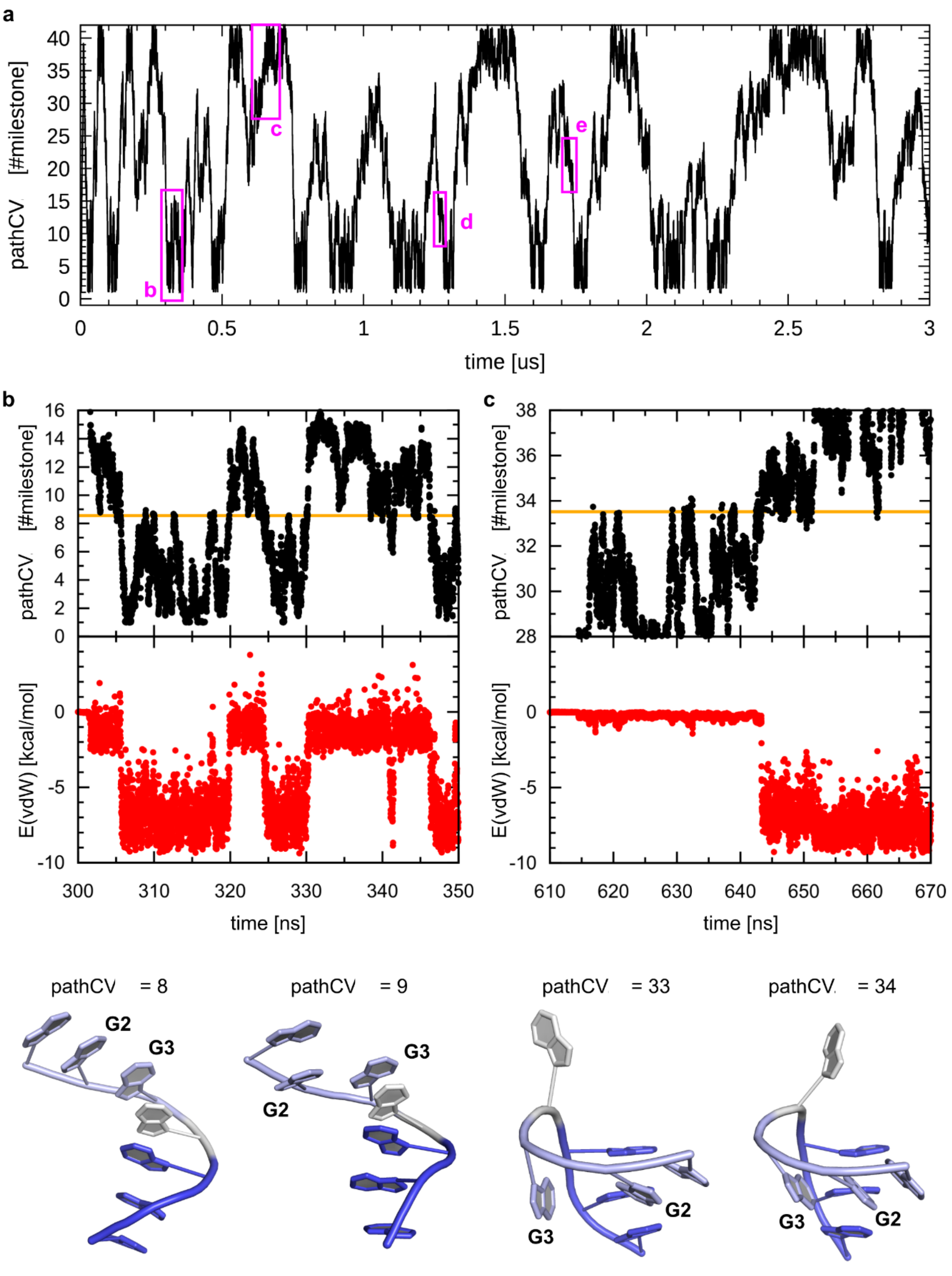

(Figure continues on next page)

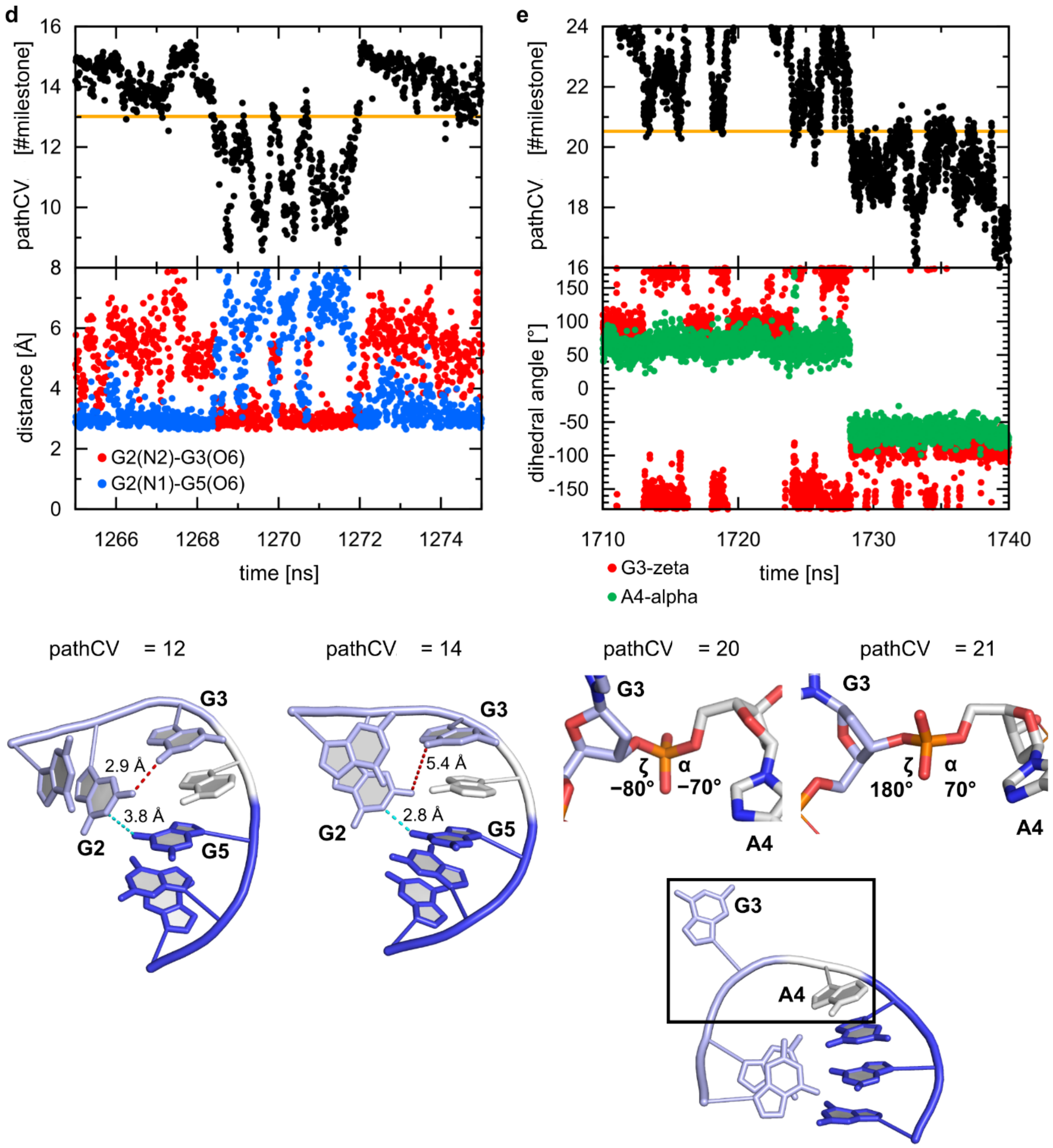

Figure S6: Sampling of the conformational space and hidden variables in the WT-MetaD simulation of the 7-mer (s→H), utilizing an RMSD-based pathCV and 42 milestones (Selection1): a) The pathCV evolution over time, b) The stacking energy of G2 and G3 bases around milestones #8 c) The stacking energy of G2 and G3 bases around milestones #33 d) Distances between G2(N2)-G3(O6) and G2(N1)-G5(O6) H-bonding interactions, e) Two dihedral angles of sugar-phosphate backbone.

WT-MetaD simulation of 7-mer based on εRMSD/RMSD pathCV

In an attempt to address the issue of hidden variables, we opted for a combination of RMSD with εRMSD for pathCV. This alteration resulted in nearly twice the number of transitions between the unfold-native-unfold states, totaling 10 turns. However, despite this adjustment, convergence of the simulation was still not achieved within the 3 μs timescale (Figure S7). The system continued to encounter difficulty navigating the conformational space (Figure S8).

Unfortunately, all previously identified hidden variables persisted, leading to the system becoming trapped in certain conformations for nanosecond timescales (Figure S8b-e). Consequently, in an effort to overcome this ongoing challenge, we decided to enhance sampling by combining WT-MetaD with REST2 within the framework of Solute-Tempering with Metadynamics.

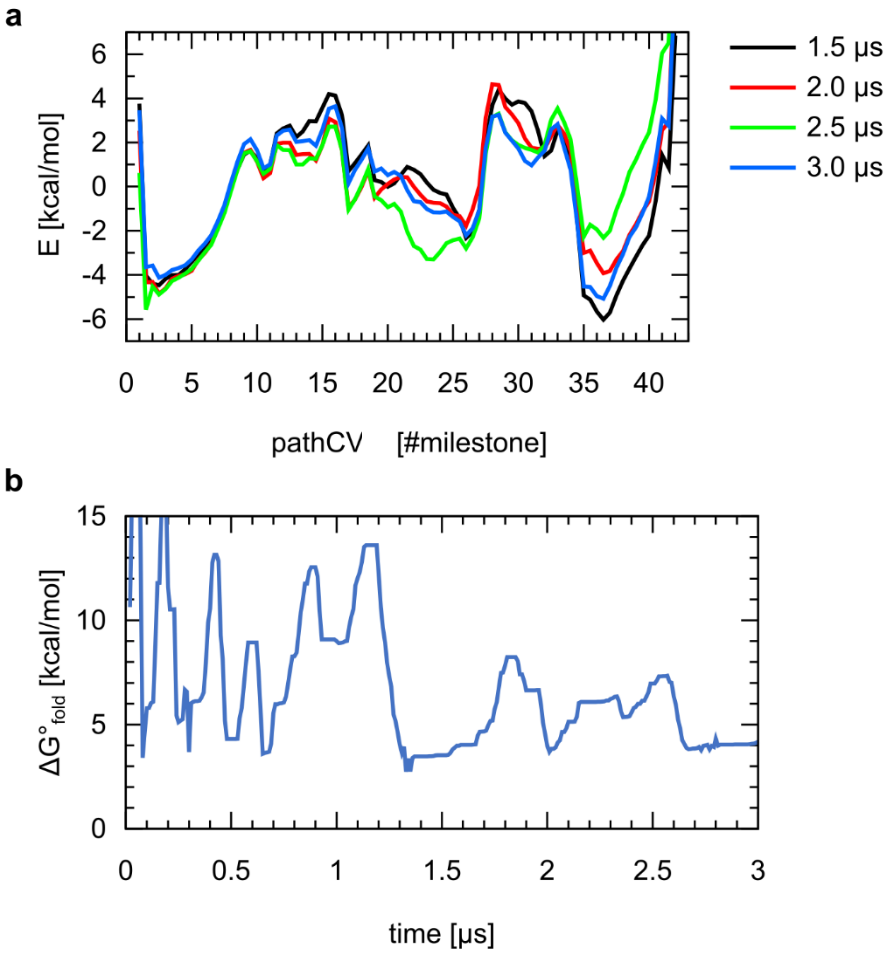

Figure S7 – Convergence of WT-MetaD (s→H) of the 7-mer with a pathCV based on εRMSD and RMSD a) FEP calculated at different time intervals b) Development of the folding energy of G-hairpin $\Delta G°_{fold}$ over time calculated using same approach as on Fig S5

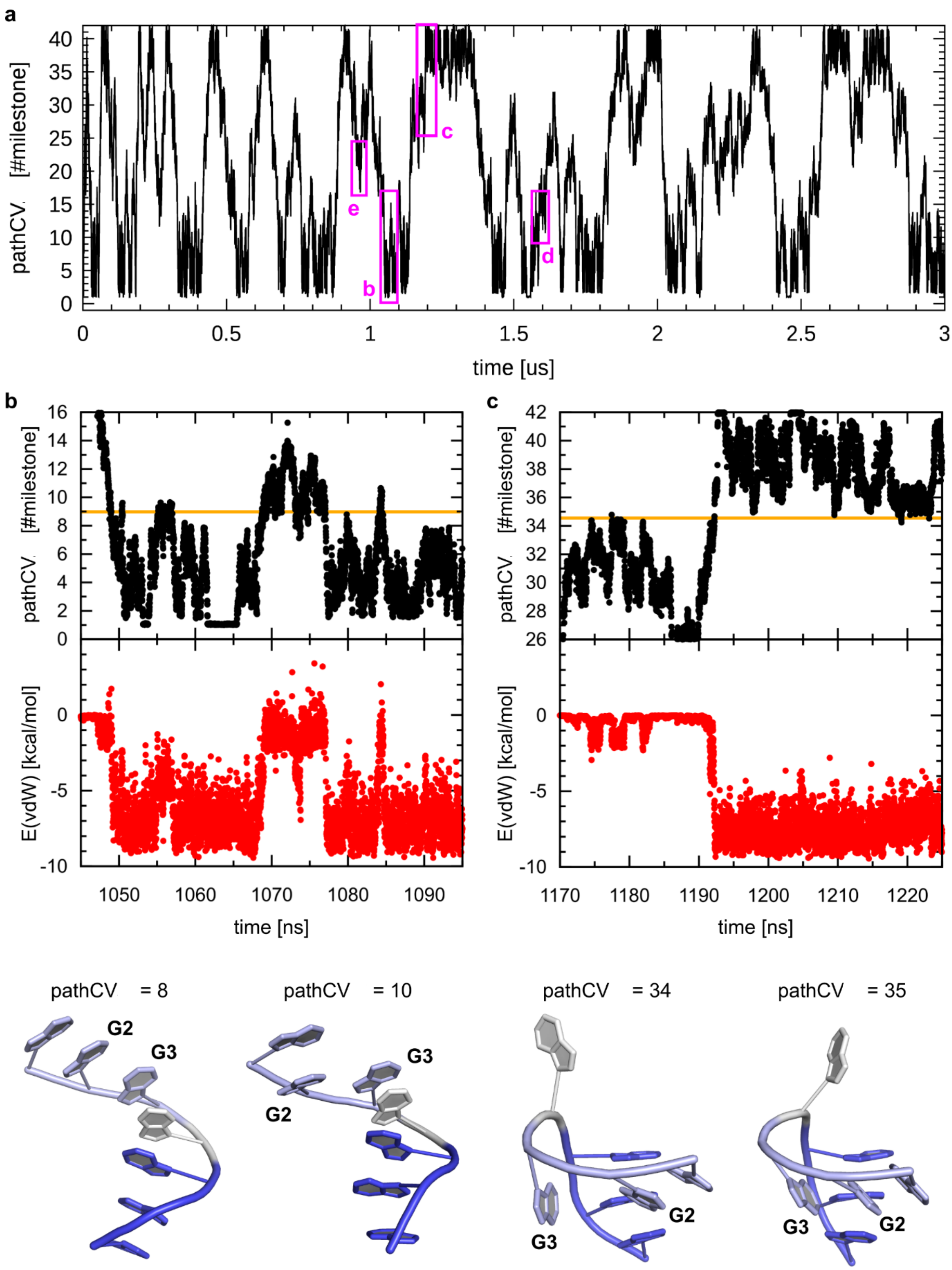

(Figure continues on next page)

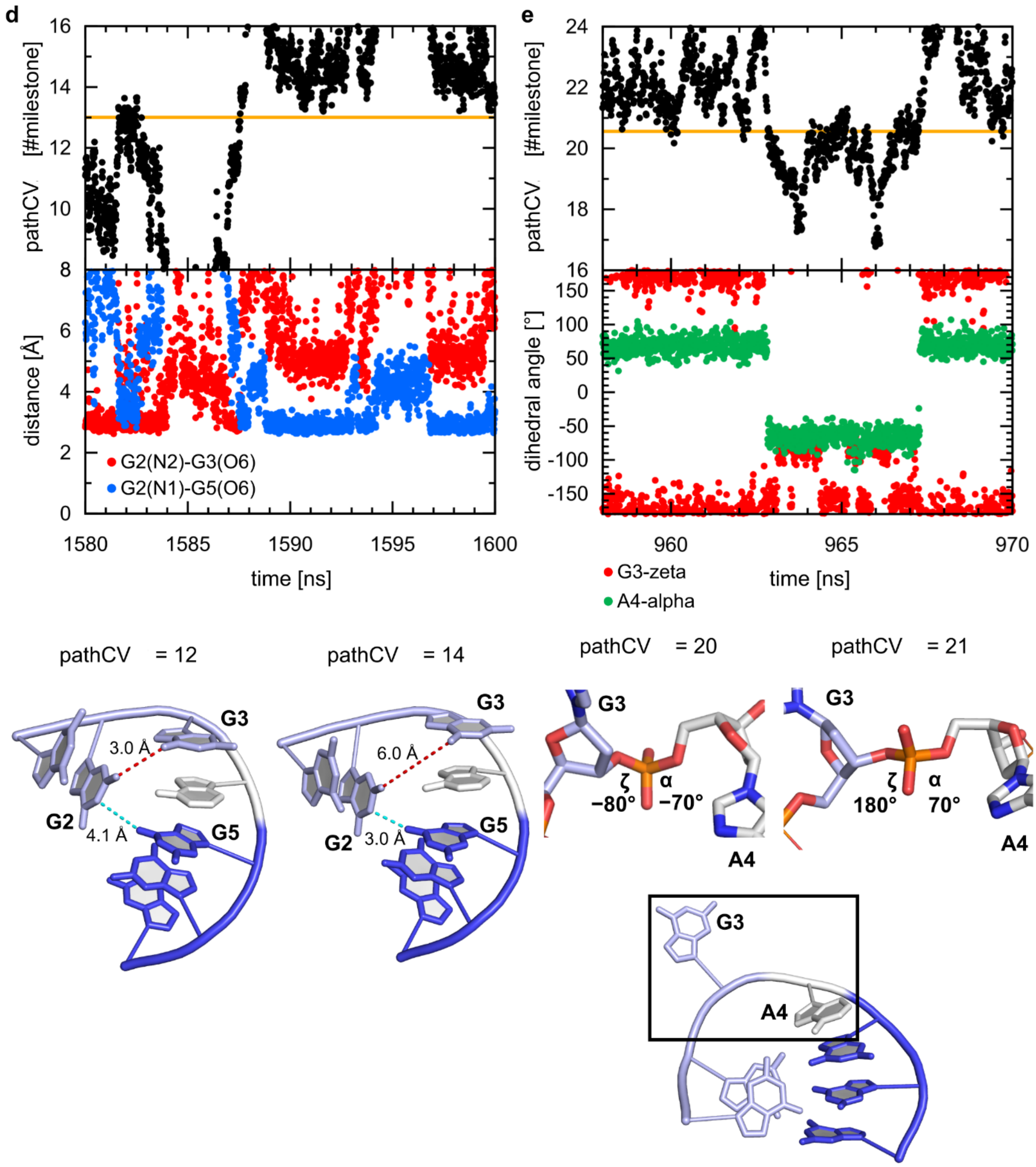

Figure S8: Sampling of the conformational space and hidden variables in the WT-MetaD simulation of the 7-mer (s→H), utilizing an εRMSD+RMSD-based pathCV and 42 milestones (Selection2) a) The pathCV evolution over time, highlighting regions where the system encountered stuck (in magenta). A correlation was observed between problematic segments of the pathCV and specific variables: b) The stacking energy of G2 and G3 bases around milestones #9 c) The stacking energy of G2 and G3 bases around milestones #33, d) Distances between G2(N2)-G3(O6) and G2(N1)-G5(O6) H-bonding interactions, e) Two dihedral angles of sugar-phosphate backbone.

ST-MetaD simulations

ST-MetaD (s→H) of the 7-mer

The combination of pathCV based on εRMSD and RMSD with ST-MetaD sampling finally resulted in simulations with sufficient convergence (see Figure S9-11).

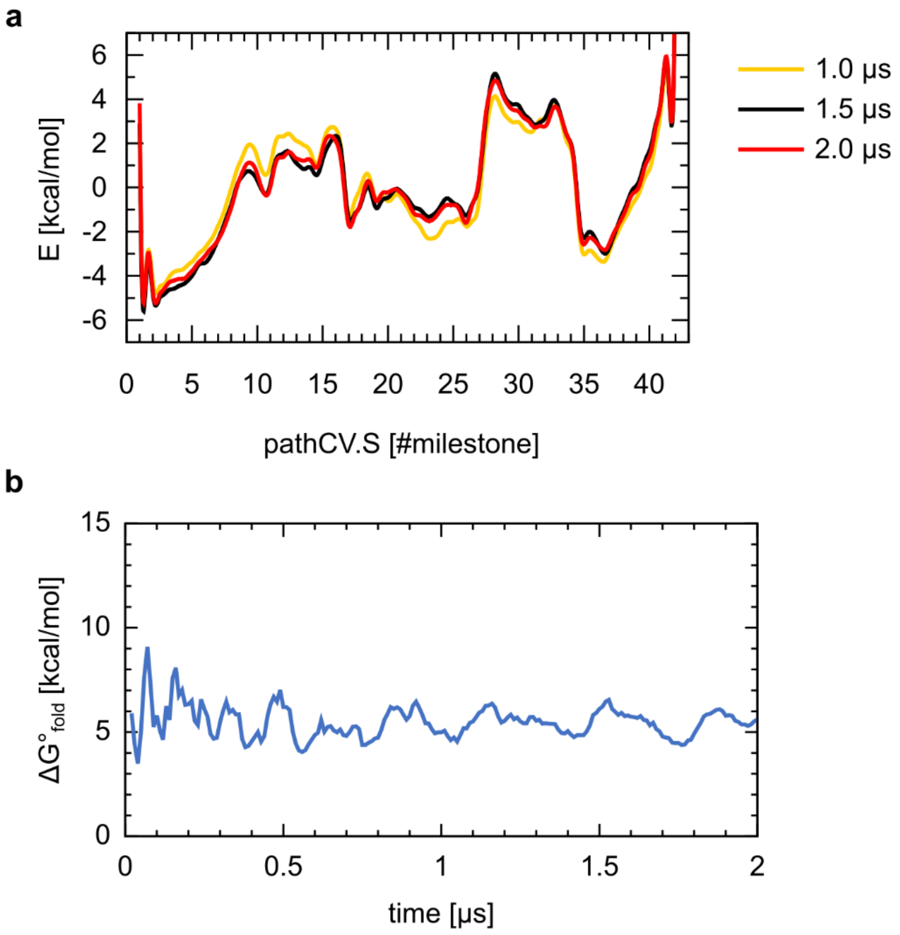

Figure S9: The convergence of the ST-MetaD (s→H) simulation of the 7-mer (refer to the list of simulations in Table S1). **a)** FEPs calculated at various time intervals **b)** The progression of folding energy of the G-hairpin, represented as $\Delta G°_{fold}$ over time. $\Delta G°_{fold}$ is calculated using the formula: $\Delta G°_{fold} = -RT[\ \ln(\Sigma_{i,native}\ w_i) - \ln(\Sigma_{i,other}\ w_i)\ ]$, where the data from the basic replica, i.e., the one running with unscaled Hamiltonian, is considered.

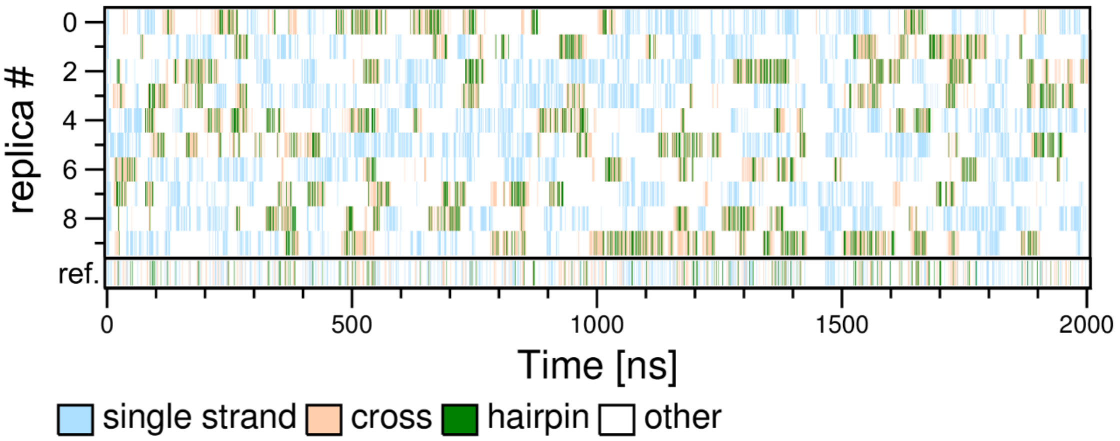

Figure S10: Conformational sampling in the ST-MetaD simulation (s→H) of the 7-mer is depicted in the panel, illustrating the time evolution of major conformers across all ten continuous (demultiplexed) replicas and reference replica (298 K). Conformers were categorized based on pathCV where: (i) Single-strand conformations correspond to values between 1.5 to 6.5, (ii) Cross conformations correspond to values between 34.5 to 39, and (iii) Native states correspond to values between 39 to 41.5. Moreover, the native state is characterized by an εRMSD bellow 0.7 to the native conformation from the 2LEE NMR structure.

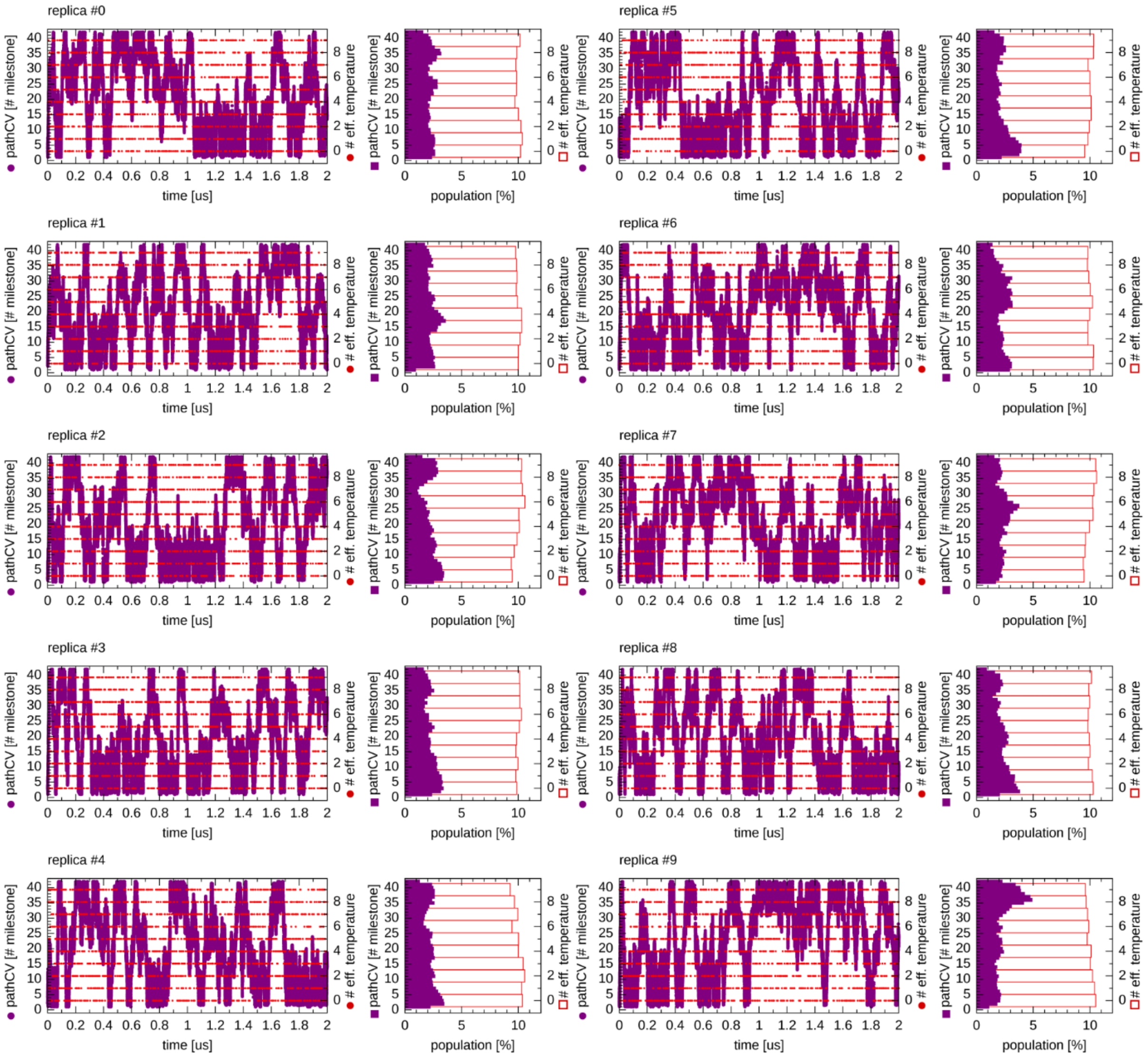

Figure S11: The time evolution of pathCV (in purple) and effective temperature (in red) across ten continuous (demultiplexed) replicas of ST-MetaD simulation of 7-mer performed on pathCV (s→H). The time evolution of pathCV is depicted with a stride of 10 ps, while the effective temperature is displayed with stride 1 ns.

Table S4: The values of the free energy profile of folding pathway of the 7-mer, calculated by weighted histogram analysis of the ST-MetaD simulation (s→H), presented alongside the standard error calculated using block averaging (Figure 3A of the main text).

PathCV	ΔE [kcal/mol]	Std. error [kcal/mol]
1.0	3.077	0.124
1.5	2.069	0.096
2.0	0.574	0.052
2.5	0.3	0.056
3.0	0.739	0.056
3.5	0.978	0.049
4.0	1.073	0.049
4.5	1.187	0.054
5.0	1.441	0.049

5.5	1.799	0.047
6.0	2.151	0.045
6.5	2.528	0.049
7.0	2.914	0.053
7.5	3.553	0.040
8.0	4.385	0.027
8.5	5.326	0.024
9.0	6.133	0.027
9.5	6.364	0.035
10.0	5.853	0.028
10.5	5.152	0.031
11.0	5.286	0.037
11.5	6.494	0.035
12.0	6.78	0.033
12.5	6.958	0.033
13.0	6.655	0.029
13.5	6.581	0.034
14.0	6.476	0.040
14.5	6.26	0.047
15.0	6.825	0.042
15.5	7.615	0.031
16.0	7.397	0.046
16.5	5.622	0.049
17.0	3.655	0.039
17.5	4.057	0.029
18.0	4.734	0.031
18.5	5.458	0.044
19.0	4.714	0.041
19.5	4.991	0.028
20.0	5.092	0.048
20.5	5.193	0.047
21.0	5.094	0.046
21.5	4.673	0.047
22.0	4.212	0.052
22.5	3.943	0.052
23.0	3.786	0.048
23.5	3.844	0.047
24.0	4.244	0.069
24.5	4.443	0.071
25.0	4.433	0.070
25.5	4.176	0.073
26.0	3.778	0.063
26.5	4.531	0.066
27.0	5.624	0.072
27.5	8.349	0.060
28.0	9.924	0.053
28.5	9.937	0.067
29.0	9.261	0.065

29.5	8.93	0.054
30.0	8.744	0.058
30.5	8.509	0.072
31.0	8.273	0.072
31.5	8.133	0.079
32.0	8.18	0.044
32.5	8.701	0.081
33.0	8.855	0.059
33.5	8.095	0.041
34.0	7.059	0.045
34.5	3.976	0.031
35.0	2.777	0.048
35.5	2.967	0.060
36.0	2.734	0.063
36.5	2.442	0.062
37.0	2.739	0.062
37.5	3.504	0.067
38.0	4.027	0.050
38.5	4.79	0.068
39.0	5.219	0.056
39.5	5.913	0.053
40.0	6.741	0.051
40.5	7.955	0.068
41.0	9.669	0.076
41.5	10.562	0.148
42.0	10.973	0.209

ST-MetaD simulations of 7-mer performed on orthogonal distance to transition path

The ST-MetaD (s→H) simulation of the 7-mer (Supplementary Table S1 and Figure 3 in the main text) involved restraining the orthogonal distance to the transition path (so called Z-coordinate), maintaining the system at orthogonal distance from pathCV transition path below 0.81 $Å^2$. However, this restraint affected the equilibrium among the single-stranded, G-hairpin, and G-cross states by limiting the available conformation space, thereby impacting the entropy the corresponding minima.

To assess the free energy between these states, we conducted two additional ST-MetaD simulations sampling the space along the orthogonal distance (Z-coordinate) from pathCV around the single-stranded (pathCV≤7) and G-cross states (pathCV≥34), see Supplementary methods. From these two ST-MetaD simulations, we calculated the free energy corrections associated with releasing the Z coordinate restraint (more specifically associated with shifting the restraint from 0.81 $Å^2$ to sufficiently large value of 4.0 $Å^2$, see Supplementary methods. The free energy corrections for single strand and G-cross states were thus determined to be 0.3 ± 0.03 kcal/mol and 3.1 ± 0.1 kcal/mol, respectively (see Figure S12).

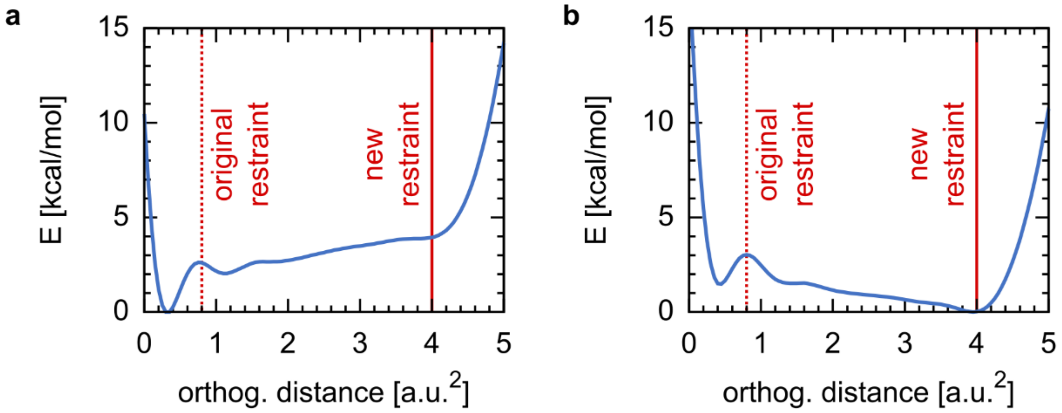

Figure S12: FEPs of two ST-MetaD simulations performed on orthogonal distance to transition path (Z coordinate), sampling conformational space around **a)** single-stranded (pathCV≤7) and **b)** G-cross (pathCV≥34) states.

The 11-mer

Structural dynamics of GGGaGGGaGGG 11-mer was described by nine simulations, two classical MD simulations, four NEB simulations, and three ST-MetaD, see Table S5.

Table S5: Summary of all performed simulations for the 11-mer.

Conformation[a]	Method	Length [μs]	Replicas	Milestones	Starting structures
ss	MD	0.1	-	-	single strand (NAB)
CC	MD	0.1	-	-	triplex-like structure (2D ST-MetaD)
ss→HH	2D ST-MetaD[b]	2	16	2x40	2x NEB s→H (selection2)
ss→sC	NEB	0.14	36	-	2D ST-MetaD ss→sC
sC→CC	NEB	0.48	40	-	2D ST-MetaD sC→CC
ss→Cs	NEB	0.14	36	-	2D ST-MetaD ss→Cs part
Cs→CC	NEB	0.2	40	-	2D ST-MetaD Cs→CC part
ss→sC→CC	ST-MetaD[b]	2	16	75	NEB ss→sC + NEB sC→CC
ss→Cs→CC	ST-MetaD[b]	2	16	75	NEB ss→Cs + NEB Cs→CC

[a] Abbreviations of conformations: s (single strand), H (hairpin), C (cross structure), s→H (path from single strand to hairpin), … etc. For 3D visualization of conformations see Figures 3, 4 and 6 in main text. **sss**→Css (bold means conformation was used for ST-MetaD with restrained pathCV)

[b] Simulations based on RMSD/εRMSD pathCV

2D ST-MetaD (ss→HH)

Table S6 – The free energy profiles of the ss->Cs->G-triplex minimum energy path, which was extracted from the 2D FES. This surface was calculated using weighted histogram analysis of the ST-MetaD_2D simulation (see Figure 4 and 5A in the main text). Error bars representing the standard error calculated using block average analysis.

Path CV1	Path CV2	index	norm. index	ΔE [kcal/mol]	Std. error [kcal/mol]
1	2	1	0.000	2.625	0.115
2	2	2	0.012	0.177	0.292
3	2	3	0.025	0.000	0.177
4	2	4	0.037	0.733	0.127
5	2	5	0.050	0.961	0.118
5	3	6	0.062	1.204	0.138
6	3	7	0.075	1.597	0.101
7	3	8	0.087	2.575	0.105
8	3	9	0.100	4.209	0.093
9	3	10	0.112	6.079	0.094
10	3	11	0.125	5.737	0.128
11	3	12	0.137	5.581	0.105
12	3	13	0.150	7.158	0.195
13	3	14	0.162	6.422	0.089
14	3	15	0.175	6.123	0.124
15	3	16	0.187	6.688	0.154
16	3	17	0.200	7.575	0.202

17	3	18	0.212	2.053	0.170
18	3	19	0.225	3.910	0.188
19	3	20	0.237	4.397	0.153
20	3	21	0.249	5.201	0.286
21	3	22	0.262	5.516	0.225
22	3	23	0.274	4.534	0.150
23	3	24	0.287	3.670	0.106
24	3	25	0.299	3.685	0.134
25	3	26	0.312	3.335	0.139
26	3	27	0.324	3.529	0.218
27	3	28	0.337	4.579	0.184
28	3	29	0.349	9.607	0.143
29	3	30	0.362	8.753	0.159
30	3	31	0.374	8.246	0.096
31	3	32	0.387	7.787	0.181
32	3	33	0.399	7.112	0.129
33	3	34	0.412	7.848	0.292
34	3	35	0.424	4.748	0.133
35	3	36	0.437	2.716	0.141
36	3	37	0.449	1.797	0.200
37	3	38	0.462	0.933	0.235
37	4	39	0.474	1.368	0.136
37	5	40	0.487	1.309	0.128
37	6	41	0.500	2.421	0.138
37	7	42	0.513	3.235	0.091
37	8	43	0.527	5.177	0.075
37	9	44	0.540	7.003	0.277
37	10	45	0.553	8.789	0.259
37	11	46	0.566	7.657	0.823
37	12	47	0.579	5.842	0.148
37	13	48	0.592	4.724	0.417
37	14	49	0.606	5.569	0.345
37	15	50	0.619	7.914	0.109
37	16	51	0.632	10.444	0.220
37	17	52	0.645	8.022	0.256
37	18	53	0.658	11.198	0.392
37	19	54	0.671	10.793	0.534
38	19	55	0.684	10.639	0.184
39	19	56	0.698	10.950	0.156
39	20	57	0.711	11.307	0.285
39	21	58	0.724	11.132	0.131
39	22	59	0.737	11.166	0.172
39	23	60	0.750	10.880	0.176
39	24	61	0.763	9.835	0.392
39	25	62	0.776	10.023	0.177
39	26	63	0.790	10.479	0.348
39	27	64	0.803	10.506	0.183
39	28	65	0.816	15.553	0.135

39	29	66	0.829	14.461	0.514
39	30	67	0.842	13.832	0.159
39	31	68	0.855	13.083	0.110
39	32	69	0.869	12.061	0.361
39	33	70	0.882	13.644	0.463
39	34	71	0.895	10.618	0.268
39	35	72	0.908	6.986	0.164
39	36	73	0.921	5.999	0.279
39	37	74	0.934	6.114	0.237
38	37	75	0.947	4.691	0.289
38	38	76	0.961	4.866	0.455
38	39	77	0.974	6.267	0.157
39	39	78	0.987	6.477	0.191
39	40	79	1.000	6.988	0.160

Table S7 – The free energy profiles of the ss->sC->G-triplex minimum energy path extracted from the 2D FES, which was calculated using weighted histogram analysis of the ST-MetaD_2D simulation (see Figure 4 and 5B in the main text). Error bars representing the standard error calculated using block average analysis.

Path CV1	Path CV2	index	norm. index	ΔE [kcal/mol]	Std. error [kcal/mol]
3	1	1	0.000	3.375	0.227
3	2	2	0.013	0.000	0.177
3	3	3	0.026	0.416	0.125
3	4	4	0.038	0.746	0.093
3	5	5	0.051	1.440	0.093
3	6	6	0.064	1.792	0.076
3	7	7	0.077	2.911	0.106
3	8	8	0.090	3.873	0.096
3	9	9	0.102	5.327	0.091
3	10	10	0.115	5.080	0.107
3	11	11	0.128	4.982	0.099
3	12	12	0.141	5.622	0.116
3	13	13	0.154	5.593	0.115
3	14	14	0.167	5.895	0.129
3	15	15	0.179	6.402	0.198
3	16	16	0.192	7.627	0.094
3	17	17	0.205	3.733	0.100
3	18	18	0.218	4.443	0.107
4	18	19	0.231	4.843	0.119
4	19	20	0.243	4.994	0.136
4	20	21	0.256	5.021	0.142
4	21	22	0.269	5.158	0.116
4	22	23	0.282	4.521	0.134
4	23	24	0.295	4.101	0.184
4	24	25	0.307	3.196	0.453
4	25	26	0.320	2.369	0.189
4	26	27	0.333	2.529	0.190

4	27	28	0.346	3.161	0.121
4	28	29	0.359	7.186	0.111
4	29	30	0.372	7.469	0.163
4	30	31	0.384	7.550	0.228
4	31	32	0.397	7.711	0.221
3	31	33	0.410	7.535	0.191
3	32	34	0.423	7.570	0.175
3	33	35	0.436	7.323	0.147
3	34	36	0.448	5.236	0.426
3	35	37	0.461	2.240	0.173
3	36	38	0.474	1.434	0.238
3	37	39	0.485	1.194	0.214
4	37	40	0.496	1.234	0.119
5	37	41	0.507	1.743	0.160
6	37	42	0.518	2.656	0.181
7	37	43	0.529	3.769	0.198
7	36	44	0.540	3.339	0.247
8	36	45	0.551	3.763	0.358
9	36	46	0.562	6.245	0.587
10	36	47	0.573	6.232	0.306
10	37	48	0.584	6.029	0.305
11	37	49	0.595	5.853	0.168
12	37	50	0.606	6.772	0.134
13	37	51	0.616	7.943	0.454
13	38	52	0.627	7.100	0.273
13	39	53	0.638	6.183	0.163
14	39	54	0.649	4.862	0.175
14	40	55	0.660	4.366	0.161
15	40	56	0.671	9.374	0.144
16	40	57	0.682	12.792	0.333
17	40	58	0.693	10.177	0.290
18	40	59	0.704	13.508	0.232
19	40	60	0.715	13.441	0.817
20	40	61	0.726	14.823	0.962
21	40	62	0.737	14.555	0.178
22	40	63	0.748	13.653	0.266
23	40	64	0.759	13.059	0.219
24	40	65	0.770	11.906	0.446
24	39	66	0.781	10.911	0.420
25	39	67	0.792	9.634	0.144
26	39	68	0.803	10.045	0.155
26	38	69	0.814	10.196	0.127
26	37	70	0.825	9.978	0.253
27	37	71	0.836	10.603	0.245
28	37	72	0.847	14.276	0.423
29	37	73	0.858	14.433	0.113
30	37	74	0.869	13.556	0.161
31	37	75	0.879	13.617	0.256

32	37	76	0.890	13.265	0.209
33	37	77	0.901	13.773	0.379
34	37	78	0.912	12.515	0.152
35	37	79	0.923	9.449	0.140
36	37	80	0.934	7.842	0.223
37	37	81	0.945	5.879	0.206
38	37	82	0.956	4.691	0.289
38	38	83	0.967	4.866	0.455
38	39	84	0.978	6.267	0.157
39	39	85	0.989	6.477	0.191
39	40	86	1.000	6.988	0.160

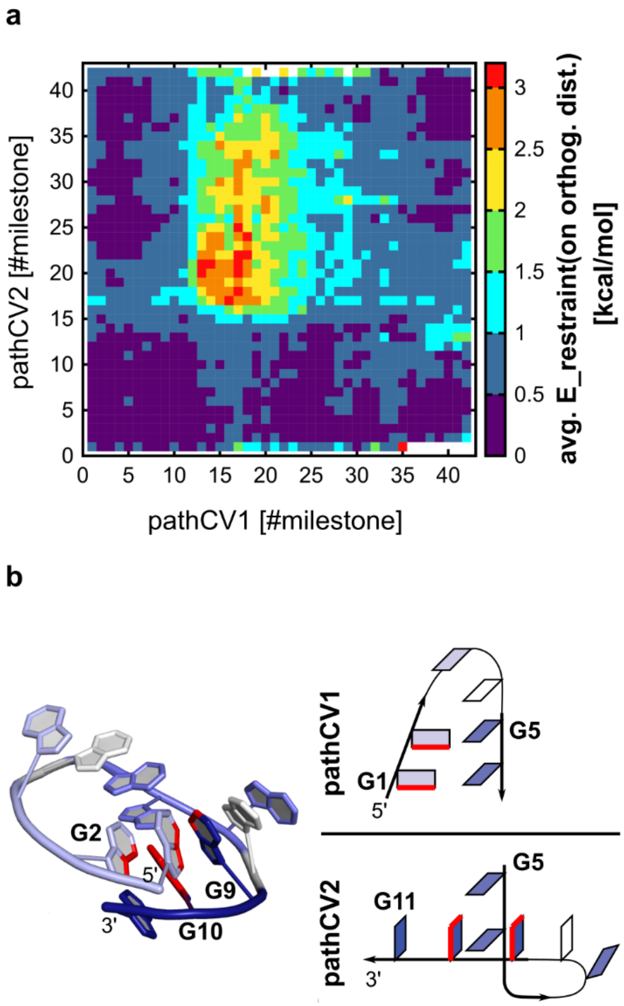

Figure S13 – a) The average bias coming from Z-coordinate restraints in 2D ST-metaD simulation indicating area, where values of pathCV1 and pathCV2 result in intramolecular clashes. b) An example of clashed structure.

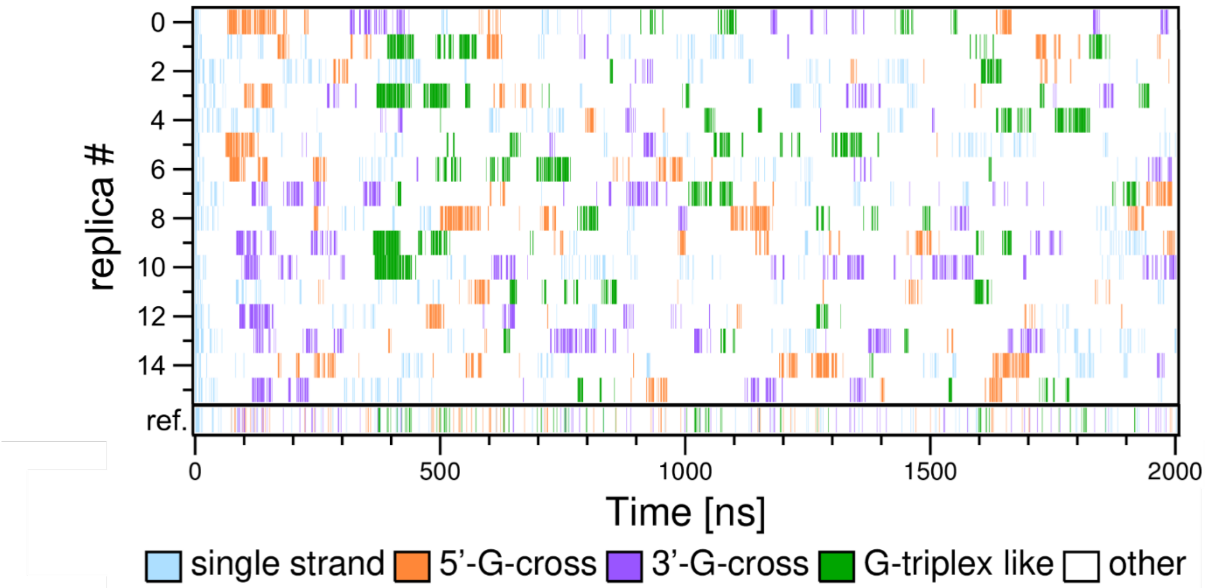

Figure S14 – Conformational sampling in the 2D ST-MetaD (ss→HH) of the 11-mer is depicted in the panel, illustrating the time evolution of major conformers across all 16 continuous (demultiplexed) trajectories and reference replica (298 K). Conformers were categorized based on pathCV1 and pathCV2 where: (i) Single strand conformations corresponds to the values between 1.5 to 6.5 for pathCV1 and between 1.5 to 6.5 for pathCV2, (ii) 5'-G-cross conformations correspond to the values between 34.5 to 41.5 for pathCV1 and between 1.5 to 6.5 for pathCV2,(iii) 3'-G-cross conformations correspond to the values between 1.5 to 6.5 for pathCV1 and between 34.5 to 41.5 for pathCV2, and (iv) G-triplex like conformations correspond to the values between 34.5 to 41.5 for pathCV1 and between 34.5 to 41.5 for pathCV2.

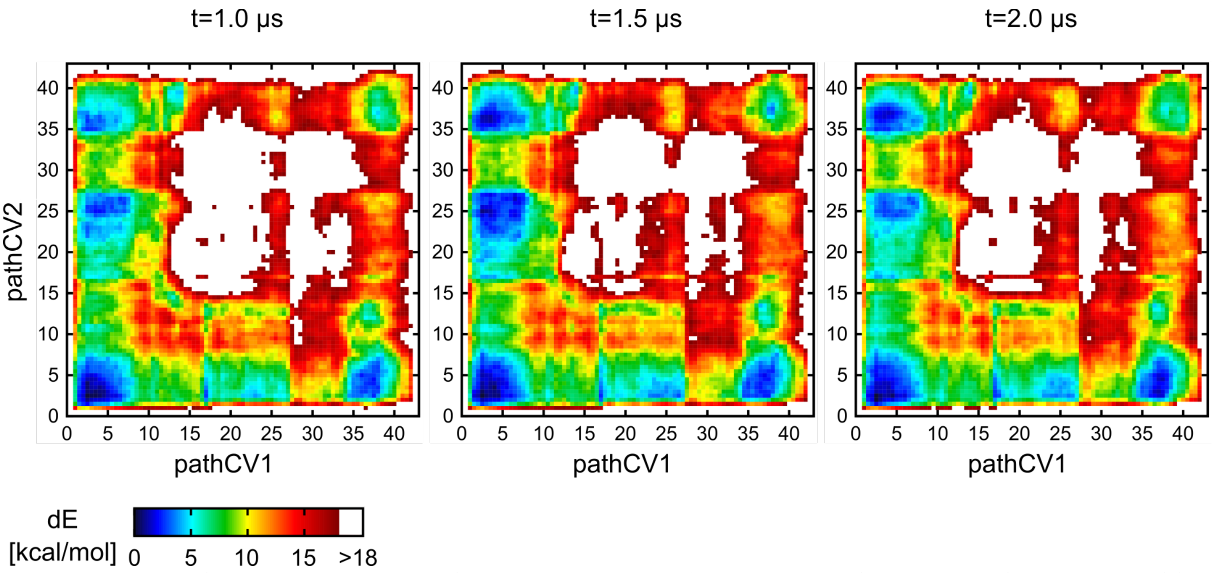

Figure S15 – The convergence of the 2D ST-MetaD (ss→HH) of the 11-mer demonstrated by the evolution of FES over time. The FES plots generated using sum_hills tool of plumed, illustrate the simulation progress at interval of 1.0; 1.5 and 2.0 µs.

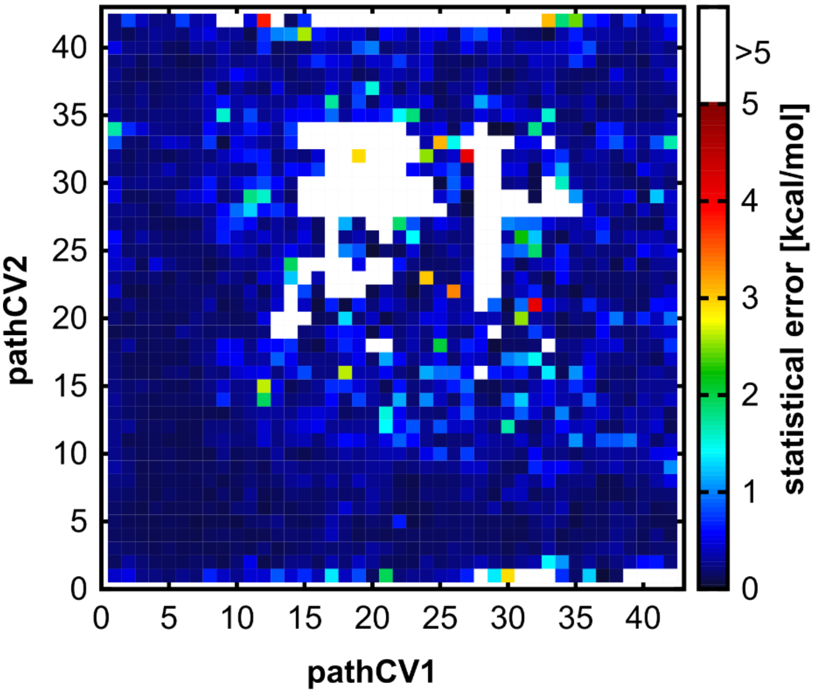

Figure S16: The statistical error associated with the free energy estimation in Figure 4 of the main text was determined using block average analysis. In certain bins of the 2D histogram, where high energy states were infrequently or not at all populated, the statistical error exceeded 30 kcal/mol. To maintain clarity, these high error values are not displayed, and instead, a uniform error of 5 kcal/mol is shown. The minimum energy pathways connecting the single stranded, G-hairpin, and G-triplex states were sampled with an error of less than 2 kcal/mol.

NEB simulations for relaxation of MEPs after 2D ST-MetaD

Preparation of starting structures

For the folding of the triplex, two distinct minimum energy paths (MEPs) were identified through 2D ST-MetaD simulations of the 11-mer, each based on a different pathCV (see Tables S6, S7, and Figure 4 for detailed MEPs and pathCV). Each MEP delineates a unique aspect of the triplex folding process, comprising 75 conformations extracted from the 2D ST-MetaD dataset. These conformations served as direct input structures for the subsequent NEB calculations.

Additionally, two equilibrated endpoints—representing the single strand and the native triplex—were included before the NEB process. Prior to NEB, these endpoints underwent a 100-ns simulation to ensure equilibration (Table S5).

Each of MEP was optimized by two independent NEB simulations, so that each NEB simulation optimized the folding of one loop, which were subsequently optimized by NEB, e.g. ss->sC and sC->G-triplex. The milestones obtained by NEB optimization were subsequently utilized in two subsequent ST-MetaD simulations.

ST-MetaD ss→Cs→CC

Table S8 – The free energy profile of the ss→Cs→CC NEB-optimized minimum energy path of the 11-mer, calculated through weighted histogram analysis of the ST-MetaD (ss→Cs→CC) simulation (see Figure 5A of the main text). Additionally, the standard error is included, calculated through block analysis.

index	norm. index	ΔE [kcal/mol]	Std. error [kcal/mol]
1	0.000	5.310	0.281
2	0.013	3.316	0.188
3	0.026	0.943	0.168
4	0.039	0.000	0.156
5	0.053	0.144	0.136
6	0.066	1.484	0.146
7	0.079	3.599	0.169
8	0.092	5.535	0.160
9	0.105	5.113	0.159
10	0.118	5.682	0.191
11	0.132	5.693	0.156
12	0.145	5.273	0.148
13	0.158	7.425	0.122
14	0.171	10.948	0.119
15	0.184	9.970	0.110
16	0.197	8.250	0.125
17	0.211	7.266	0.122
18	0.224	6.097	0.146
19	0.237	6.030	0.134
20	0.250	5.744	0.120
21	0.263	4.383	0.116
22	0.276	3.803	0.111
23	0.289	4.645	0.138
24	0.303	-0.010	0.117
25	0.316	-1.370	0.105
26	0.329	-2.617	0.075
27	0.342	-3.029	0.091
28	0.355	0.259	0.071
29	0.368	0.152	0.089
30	0.382	0.127	0.100
31	0.395	0.614	0.102
32	0.408	1.395	0.102
33	0.421	1.127	0.076
34	0.434	0.797	0.090
35	0.447	1.181	0.093
36	0.461	1.515	0.078
37	0.474	1.272	0.073
38	0.487	1.025	0.084
39	0.500	1.040	0.087

40	0.513	1.411	0.074
41	0.526	1.289	0.058
42	0.539	1.962	0.059
43	0.553	5.144	0.057
44	0.566	8.047	0.101
45	0.579	8.413	0.096
46	0.592	6.190	0.085
47	0.605	4.953	0.084
48	0.618	3.248	0.086
49	0.632	1.182	0.093
50	0.645	0.963	0.125
51	0.658	4.019	0.134
52	0.671	5.381	0.171
53	0.684	4.407	0.096
54	0.697	3.639	0.084
55	0.711	2.120	0.090
56	0.724	1.407	0.081
57	0.737	2.629	0.080
58	0.750	5.454	0.123
59	0.763	3.298	0.179
60	0.776	4.067	0.776
61	0.789	6.790	0.143
62	0.803	1.339	0.175
63	0.816	1.797	0.184
64	0.829	2.872	0.116
65	0.842	2.478	0.138
66	0.855	2.677	0.095
67	0.868	2.980	0.114
68	0.882	3.815	0.142
69	0.895	4.101	0.163
70	0.908	3.712	0.067
71	0.921	2.494	0.107
72	0.934	2.206	0.144
73	0.947	3.311	0.225
74	0.961	3.249	0.279
75	0.974	3.770	0.329
76	0.987	6.047	0.202
77	1.000	9.627	1.323

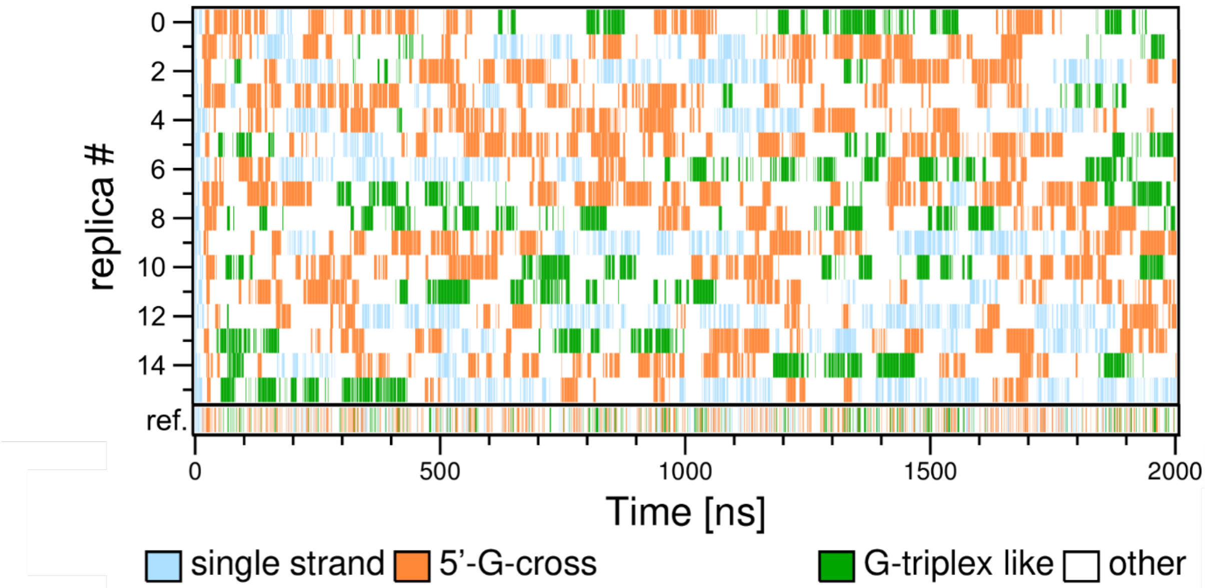

Figure S17 - Conformational sampling in the ST-MetaD (ss→Cs→CC) of the 11-mer is depicted in the panel, illustrating the time evolution of major conformers across all 16 continuous (demultiplexed) trajectories and reference replica (298 K). Conformers were categorized based on pathCV where: (i) Single strand conformations correspond to values between 1.5 to 6.5, (ii) 5'-G-cross conformations correspond to values between 28 to 42.5, and (iii) G-triplex like conformations correspond to values between 69.5 to 76.5.

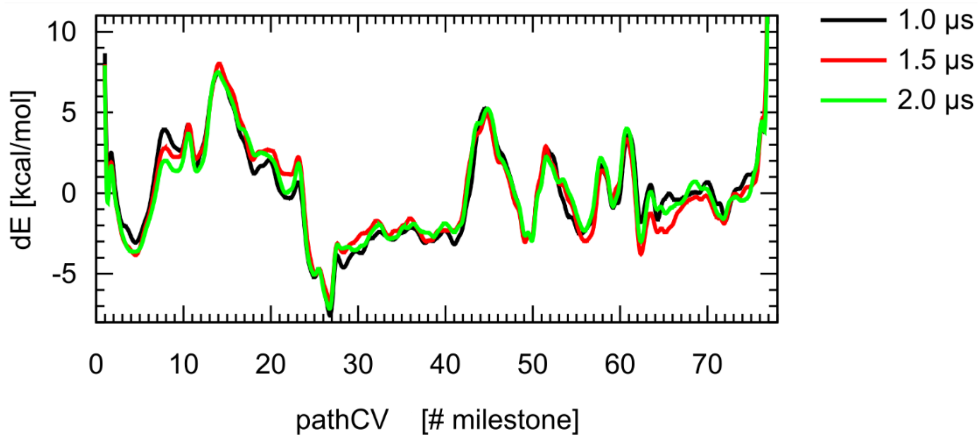

Figure S18 – The convergence of ST-MetaD (ss→Cs→CC) of the 11-mer is depicted through the time evolution of the free energy profile (FEP) after 1.0, 1.5, and 2.0 μs of ST-MetaD. These profiles were calculated using the sum hills tool of plumed.

ST-MetaD ss→sC→CC

Table S9 - The free energy profile of the ss→sC→CC NEB-optimized minimum energy path of the 11-mer, calculated through weighted histogram analysis of the ST-MetaD (ss→sC→CC) simulation (see Figure 5B of the main text). Additionally, the standard error is included, calculated through block analysis.

index	norm. index	ΔE [kcal/mol]	Std. error [kcal/mol]
1	0.000	4.493	0.495
2	0.013	1.476	0.144
3	0.026	0	0.150
4	0.039	0.029	0.173
5	0.053	0.052	0.168
6	0.066	0.094	0.138
7	0.079	0.703	0.139
8	0.092	1.597	0.160
9	0.105	2.608	0.160
10	0.118	4.439	0.165
11	0.132	5.263	0.147
12	0.145	5.933	0.141
13	0.158	6.948	0.347
14	0.171	7.384	0.268
15	0.184	7.116	0.247
16	0.197	7.761	0.252
17	0.211	8.615	0.140
18	0.224	8.433	0.137
19	0.237	11.825	0.122
20	0.250	10.002	0.088
21	0.263	7.687	0.074
22	0.276	6.739	0.098
23	0.289	6.485	0.118
24	0.303	7.069	0.111
25	0.316	8.237	0.103
26	0.329	6.512	0.076
27	0.342	4.515	0.072
28	0.355	3.897	0.098
29	0.368	5.124	0.097
30	0.382	8.378	0.138
31	0.395	7.711	0.113
32	0.408	4.25	0.135
33	0.421	1.543	0.112
34	0.434	0.844	0.095
35	0.447	0.323	0.098
36	0.461	-0.062	0.090
37	0.474	-0.186	0.081
38	0.487	0.227	0.075
39	0.500	0.968	0.077

40	0.513	0.864	0.086
41	0.526	0.449	0.087
42	0.539	0.366	0.080
43	0.553	0.758	0.092
44	0.566	1.478	0.106
45	0.579	3.201	0.119
46	0.592	3.55	0.102
47	0.605	3.839	0.108
48	0.618	1.317	0.140
49	0.632	1.016	0.111
50	0.645	1.314	0.119
51	0.658	2.555	0.134
52	0.671	5.92	0.100
53	0.684	7.371	0.121
54	0.697	7.218	0.080
55	0.711	5.453	0.183
56	0.724	5.823	0.629
57	0.737	5.9	0.311
58	0.750	6.236	0.313
59	0.763	7.719	0.443
60	0.776	8.99	0.123
61	0.789	8.23	0.078
62	0.803	8.937	0.056
63	0.816	8.97	0.193
64	0.829	9.211	0.148
65	0.842	11.98	0.166
66	0.855	12.177	0.158
67	0.868	10.516	0.176
68	0.882	10.811	0.331
69	0.895	11.195	0.346
70	0.908	11.043	0.173
71	0.921	9.306	0.174
72	0.934	7.421	0.203
73	0.947	4.997	0.404
74	0.961	3.178	0.167
75	0.974	3.04	0.194
76	0.987	4.147	0.286
77	1.000	8.045	0.170

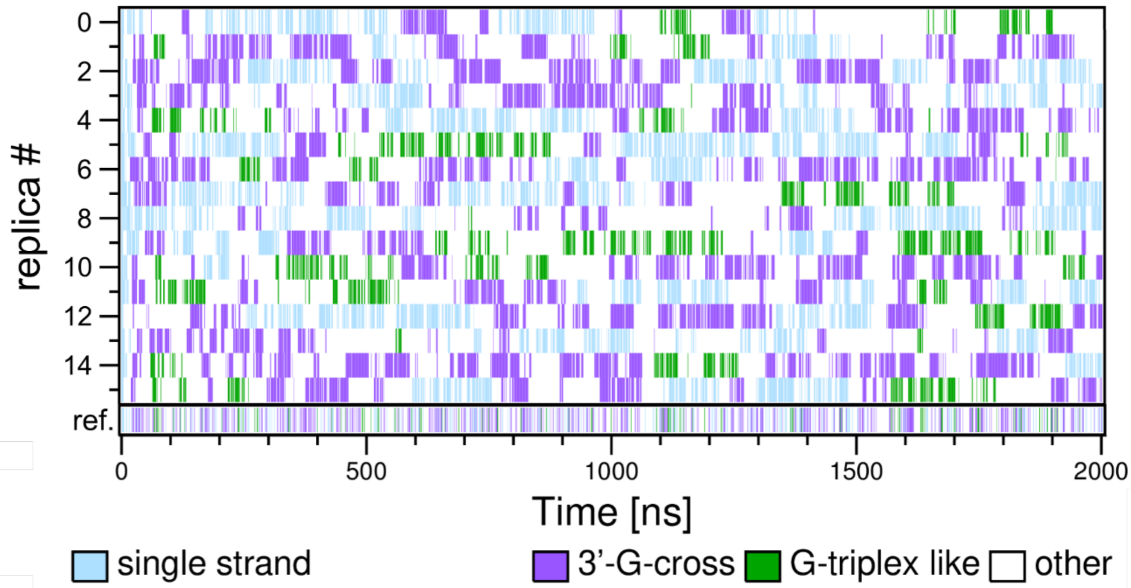

Figure S19 - Conformational sampling in the ST-MetaD simulations (ss→sC→CC) of the 11-mer, illustrating the time evolution of major conformers across all 16 continuous (demultiplexed) trajectories and reference replica (298 K). Conformers were categorized based on pathCV where: (i) Single strand conformations correspond to between 1.5 to 9, (ii) 3'-G-cross conformations correspond to values between 32.5 to 44.5, and (iii) G-triplex like conformations correspond to values between 72.5 to 76.5.

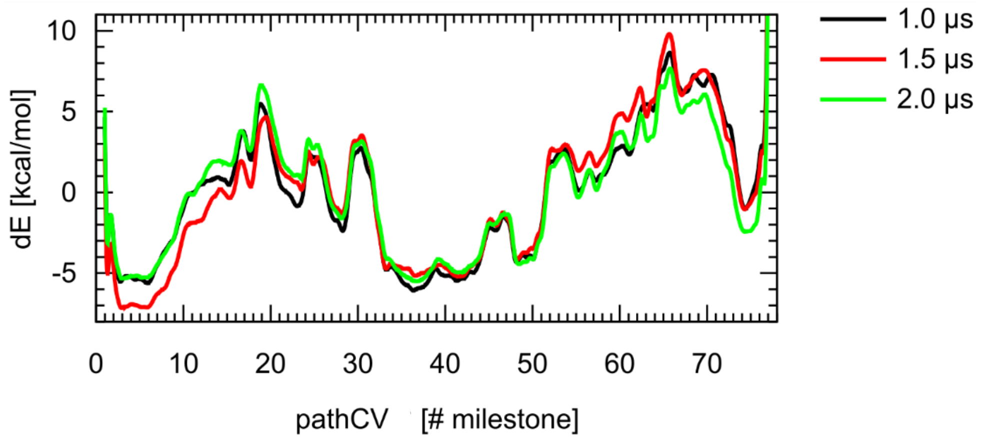

Figure S20: The convergence of ST-MetaD (ss→sC→CC) of the 11-mer is shown through the time evolution of the FEP after 1.0, 1.5, and 2.0 μs of ST-MetaD, calculated using the sum_hills tool of Plumed.

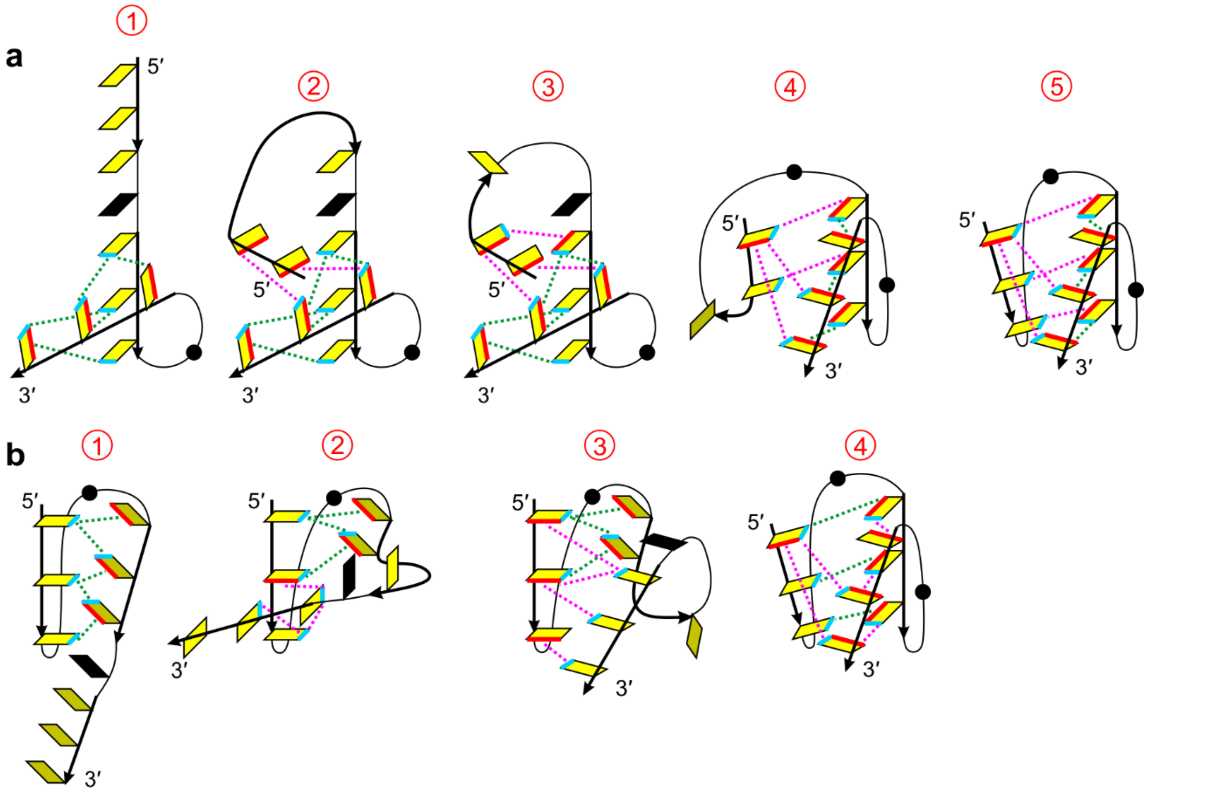

Figure S21: Intermediates of G-triplex folding, starting either from the 3' or 5'-G-cross, are illustrated in panels (a) and (b), respectively. These correspond to the FEPs shown in Figure 5 A and B in the main text, respectively. The Watson-Crick (WC) and Hoogsteen edges are depicted in blue and red, respectively. Additionally, the hydrogen bond interactions of the G-cross present at the beginning (in structure #1) are denoted by green dotted lines, while the other interactions leading to the formation of the triplex-like structure are represented by magenta dotted lines.

The 15-mer

Structural dynamics of 15-mer guanine quadruplex was described by 56 simulations, eight classical MD simulations corresponding to folding states at vertexes of the folding cube (see Figure 6A in the main text), 12 NEB simulations corresponding to transition paths along the edges of the cube, and 36 (3x12) ST-MetaD (12 for ST-MetaD along pathCVs of the edges and 2x12 of ST-MetaD simulations of Z-coordinates), see Table S10.

Table S10: Summary of all performed simulations for the 15-mer.

Conformation[a]	Method	Length [μs]	Replicas	Milestones	Starting structures
sss	MD	2	-	-	single strand (NAB)
Hss	MD	2	-	-	G-hairpin (PDB ID:2LEE) + single strand (NAB)
sHs	MD	2	-	-	G-hairpin (PDB ID:2LEE) + single strand (NAB)
ssH	MD	2	-	-	G-hairpin (PDB ID:2LEE) + single strand (NAB)
HHs	MD	2	-	-	G-triplex (PDB ID:2LEE) + single strand (NAB)

Conformation[a]	Method	Length [μs]	Replicas	Milestones	Starting structures
HsH	MD	2	-	-	G-hairpin (PDB ID:2LEE) + single strand (NAB)
sHH	MD	2	-	-	G-triplex (PDB ID:2LEE) + single strand (NAB)
HHH	MD	2	-	-	G-quadruplex (PDB ID:2LEE)
sss→Css	NEB	0.2	40	-	ref. 11
sss→sCs	NEB	0.2	40	-	ref. 11
sss→ssC	NEB	0.2	40	-	ref. 11
Css→CCs	NEB	0.2	40	-	ref. 11
Css→CsC	NEB	0.2	40	-	ref. 11
sCs→CCs	NEB	0.2	40	-	ref. 11
sCs→sHC	NEB	0.2	40	-	ref. 11
ssC→sHC	NEB	0.2	40	-	ref. 11
ssC→CsC	NEB	0.2	40	-	ref. 11
CCs→HHH	NEB	0.2	40	-	ref. 11
CsC→HHH	NEB	0.2	40	-	ref. 11
sHC→HHH	NEB	0.2	40	-	ref. 11
sss→Css	ST-MetaD[b]	2	16	40	NEB sss→Css
sss→sCs	ST-MetaD[b]	2	16	40	NEB sss→sCs
sss→ssC	ST-MetaD[b]	2	16	40	NEB sss→ssC
Css→CCs	ST-MetaD[b]	2	16	40	NEB Css→CCs
Css→CsC	ST-MetaD[b]	2	16	40	NEB Css→CsC
sCs→CCs	ST-MetaD[b]	2	16	40	NEB sCs→CCs
sCs→sHC	ST-MetaD[b]	2	16	40	NEB sCs→sHC
ssC→sHC	ST-MetaD[b]	2	16	40	NEB ssC→sHC
ssC→CsC	ST-MetaD[b]	2	16	40	NEB ssC→CsC
CCs→HHH	ST-MetaD[b]	2	16	40	NEB CCs→HHH
CsC→HHH	ST-MetaD[b]	2	16	40	NEB CsC→HHH
sHC→HHH	ST-MetaD[b]	2	16	40	NEB sHC→HHH
**sss**→Css	ST-MetaD[c]	0.5	16	#1-#7	NEB sss→Css
sss→**Css**	ST-MetaD[c]	0.5	16	#36-#42	NEB sss→Css
**sss**→sCs	ST-MetaD[c]	0.5	16	#1-#7	NEB sss→sCs
sss→**sCs**	ST-MetaD[c]	0.5	16	#36-#42	NEB sss→sCs
**sss**→ssC	ST-MetaD[c]	0.5	16	#1-#7	NEB sss→ssC
sss→**ssC**	ST-MetaD[c]	0.5	16	#36-#42	NEB sss→ssC
**Css**→CCs	ST-MetaD[c]	0.5	16	#1-#7	NEB Css→CCs
Css→**CCs**	ST-MetaD[c]	0.5	16	#36-#42	NEB Css→CCs
**Css**→CsC	ST-MetaD[c]	0.5	16	#1-#7	NEB Css→CsC
Css→**CsC**	ST-MetaD[c]	0.5	16	#36-#42	NEB Css→CsC
**sCs**→CCs	ST-MetaD[c]	0.5	16	#1-#7	NEB sCs→CCs
sCs→**CCs**	ST-MetaD[c]	0.5	16	#36-#42	NEB sCs→CCs
**sCs**→sHC	ST-MetaD[c]	0.5	16	#1-#7	NEB sCs→sHC
sCs→**sHC**	ST-MetaD[c]	0.5	16	#34-#42	NEB sCs→sHC
**ssC**→sHC	ST-MetaD[c]	0.5	16	#1-#7	NEB ssC→sHC
ssC→**sHC**	ST-MetaD[c]	0.5	16	#34-#42	NEB ssC→sHC

Conformation[a]	Method	Length [μs]	Replicas	Milestones	Starting structures
**ssC**→CsC	ST-MetaD[c]	0.5	16	#1-#7	NEB ssC→CsC
ssC→**CsC**	ST-MetaD[c]	0.5	16	#36-#42	NEB ssC→CsC
**CCs**→HHH	ST-MetaD[c]	0.5	16	#1-#7	NEB CCs→HHH
CCs→**HHH**	ST-MetaD[c]	0.5	16	#36-#42	NEB CCs→HHH
**CsC**→HHH	ST-MetaD[c]	0.5	16	#1-#7	NEB CsC→HHH
CsC→**HHH**	ST-MetaD[c]	0.5	16	#36-#42	NEB CsC→HHH
**sHC**→HHH	ST-MetaD[c]	0.5	16	#1-#9	NEB sHC→HHH
sHC→**HHH**	ST-MetaD[c]	0.5	16	#36-#42	NEB sHC→HHH

[a] Abbreviations of conformations: s (single strand), H (hairpin), C (cross structure), s→H (path from single strand to hairpin), … etc. For 3D visualization of conformations see Figures 3, 5 and 6 in main text. **sss**→Css (bold means conformation was used for ST-MetaD with restrained pathCV)…

[b] Simulations based on RMSD/εRMSD pathCV

[c] ST-MetaD performed only on Z coordinate

Classical MD simulations

As anticipated, the parallel G-triplex and parallel G-hairpin conformations surrounded by a single-stranded overhang, proved unstable for more than a few nanoseconds in classical MD simulations (Figures S22 and S23), undergoing transformation of its hairpin loops to G-crosses. In particular, both G-triplex structures (HHs, sHH) rapidly transitioned towards the HC state (HCs and sHC), where they remained stable for approximately 10 ns. Subsequently, they further unfold to G-crossed structures (CCs and sCC), see Figure S22a,b).

In the HsH state (Figure S22c), the hairpins swiftly rearranged into a tilted cross state (CsC_1), stabilized by clashes between the backbones of A4 and A12. Although this state was not highly populated, each cross had the opportunity to fluctuate back to the hairpin state. After 20 ns, the clash between A4 and A12 was released, and a G3|G13 stack was formed (CsC_2), remaining stable on the microsecond scale and thus chosen as the relevant intermediate.

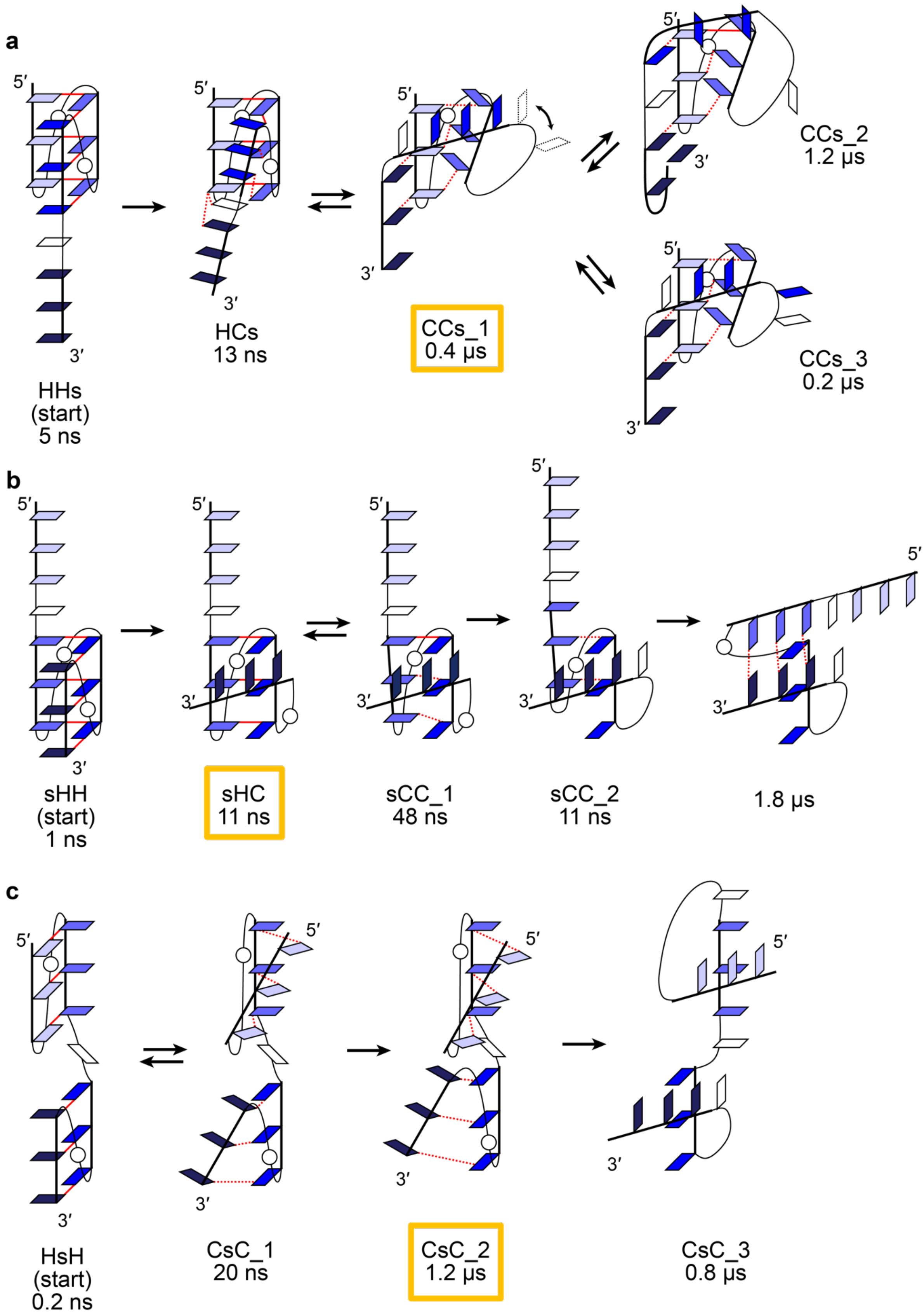

Figure S22: The stability and dynamics of putative folding intermediates of the parallel G-quadruplex investigated through classical MD simulations. The starting structures included a parallel G-triplex with a 3'-overhang (a), a triplex with a 5'-overhang (b), and a structure where two G-hairpins are connected by a single-adenine loop (c). These structures were derived from

the parallel G-quadruplex (PDB ID: 2LEE) by substituting one G-hairpin with a single-stranded A-DNA chain. The conformation of the d[(GGGA)$_3$GGG] sequence is represented by three letters, as explained in Figure 6a, where "s", "H", and "C" stand for single strand, hairpin, and cross, respectively. The coloring of the schemes is consistent with Figure 6c, and structures chosen as relevant intermediates for further study by the enhanced sampling approach are highlighted in yellow.

The single-hairpin structures (Hss, sHs, ssH) exhibited similar dynamics. The G-hairpin remained stable for no longer than 1 ns before rearranging into the G-cross state (Figure S22). The loop adenine stacked on top of the 2nd G-tract of the G-cross, stabilizing the structure for the remainder of the 2-μs-long simulation unless disturbed by misfolding of the rest of the molecule. Consequently, the one-cross structures (Css, sCs, ssC) were chosen as the relevant intermediate.

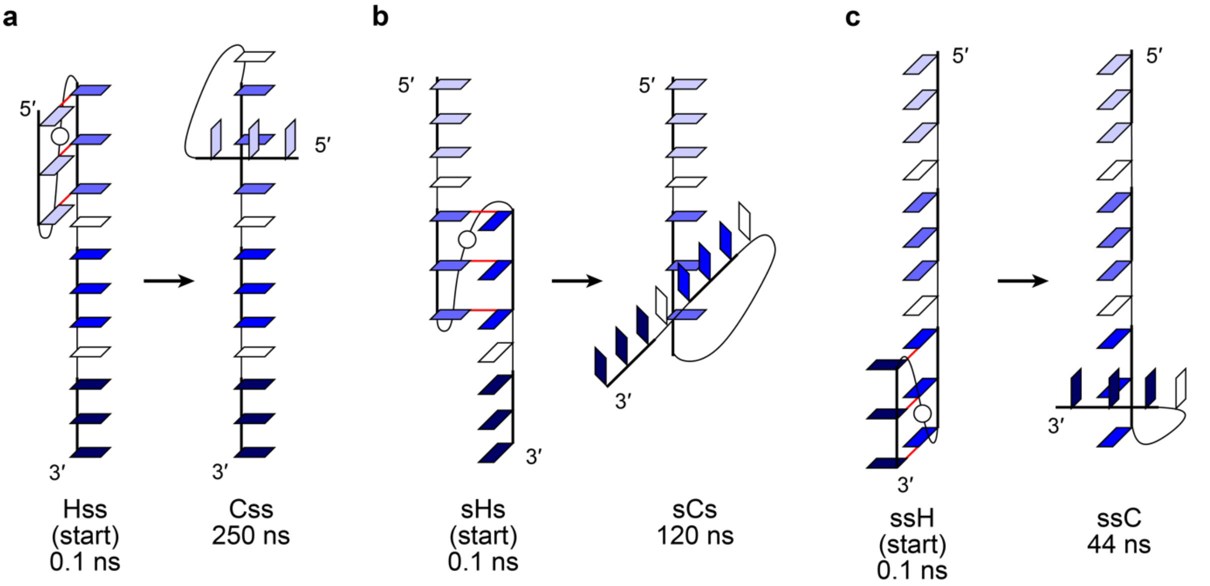

Figure S23: The stability and dynamics of putative folding intermediates of the parallel G-quadruplex examined using classical MD simulations. The starting structures consisted of single-stranded A-DNA chains with two G-tracts forming a parallel G-hairpin, positioned at the 5'-end (a), in the middle (b), or at the 3'-end (c). Upon simulation, the G-hairpin conformation rapidly rearranged into a G-cross structure, which remained stable unless disrupted by misfolding in other parts of the chain. The remaining portions of the chain remained in the single-stranded state for 44 to 250 ns of simulation time before undergoing irreversible rearrangements to form cross structures or misfolded states.

NEB simulations

Preparation of starting structures

The folding dynamics of the entire quadruplex were represented by the edges of a cube, as illustrated in Figure 6A of the main text. Each vertex of the cube represents an individual endpoint for the NEB simulation. The endpoint conformations were prepared as follows: (i) the single-stranded state was constructed using Nucleic Acid Builder (AMBER14) to mimic an B-form single strand, (ii) the parallel G-quadruplex state was modeled using a published NMR structure (PDB ID: 2LEE), and (iii) intermediate states were modeled using a combination of the 2LEE structure and the B-form single strand. Prior to the NEB simulation,

each endpoint was equilibrated by 2-μs-long MD simulation discussed in the previous chapter (Classical MD simulations). Each of these MD simulations sampled more than one conformer, and endpoints were chosen as equilibrated conformers highlighted in Figures S22 and S23. Subsequently, the evenly spaced milestones for pathCV along each edge of the cube were selected from the NEB simulations.

ST-MetaD simulations of 15-mer

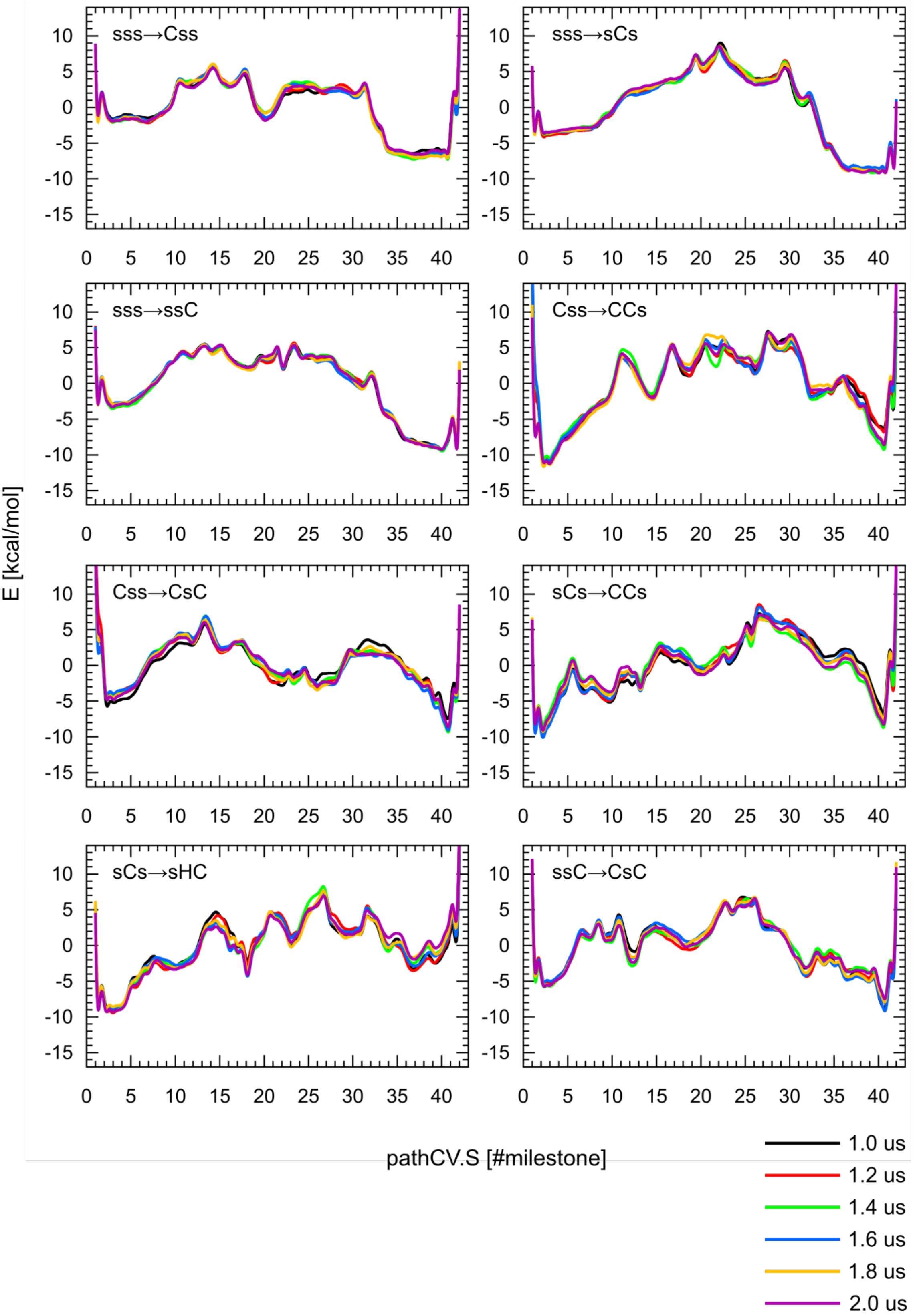

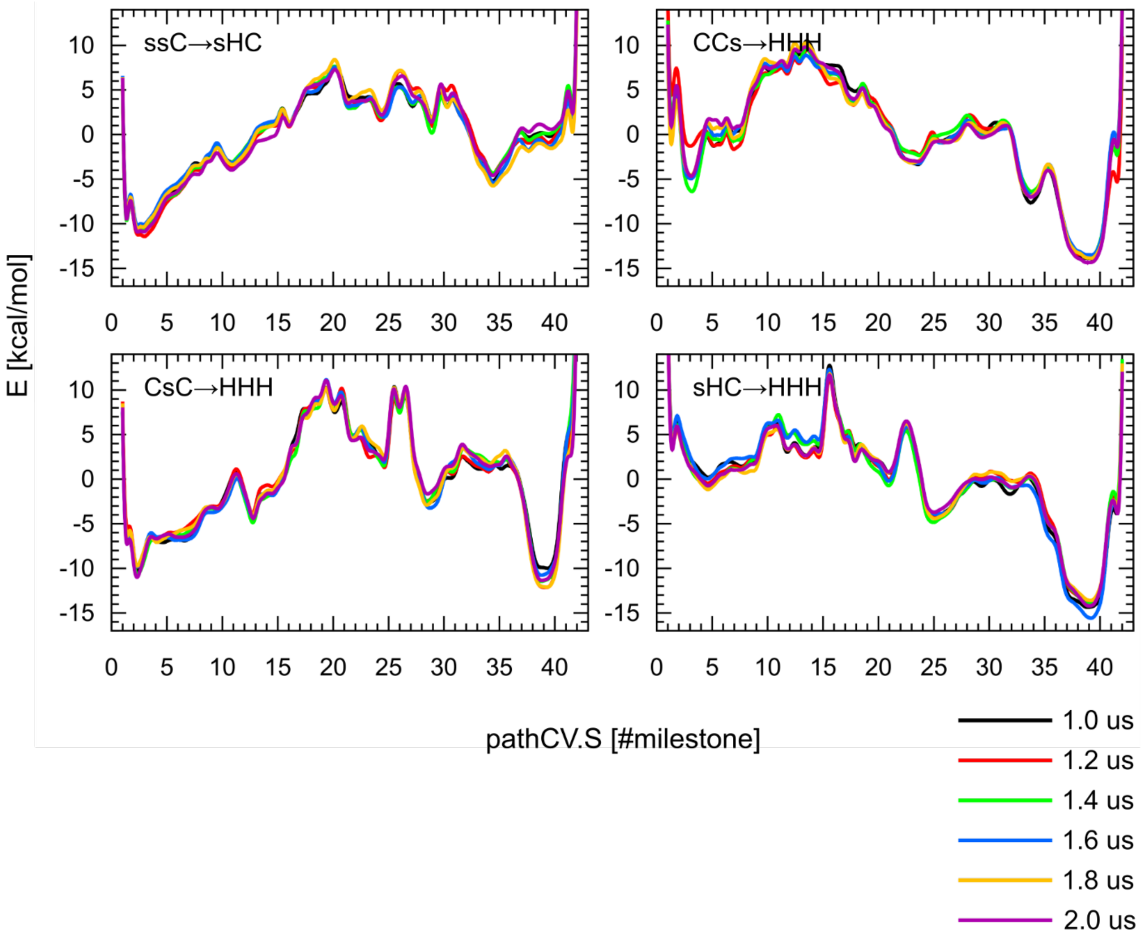

Figure S24 – Convergence of the 12 ST-MetaD simulations of the 15-mer is illustrated by the time evolution of the FEP, covering simulation times from 1.0 up to 2.0 μs. The FEPs were calculated with a stride of 0.2 μs using the sum_hills tool of Plumed.

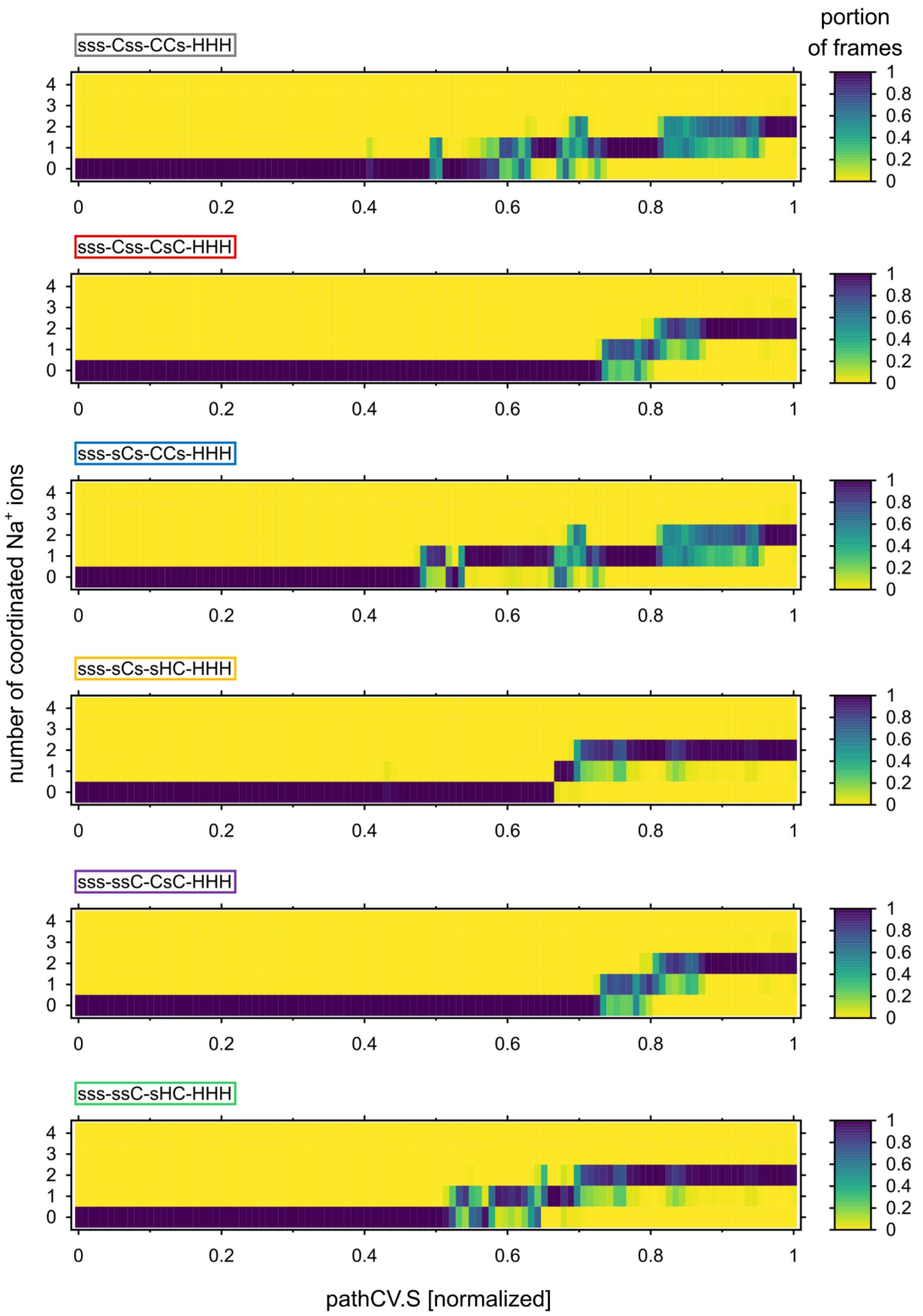

Figure S25 – The population distribution of frames depicting the number of Na+ cations within the folded or partially folded quadruplex channel across six different pathways (cf. Figure 7 of the main text). Each diagram presents the population of frames where "N" Na^+ cations were coordinated within the channel at specific values of pathCV. Na^+ cations were counted if they were coordinated by at least four O6 atoms.

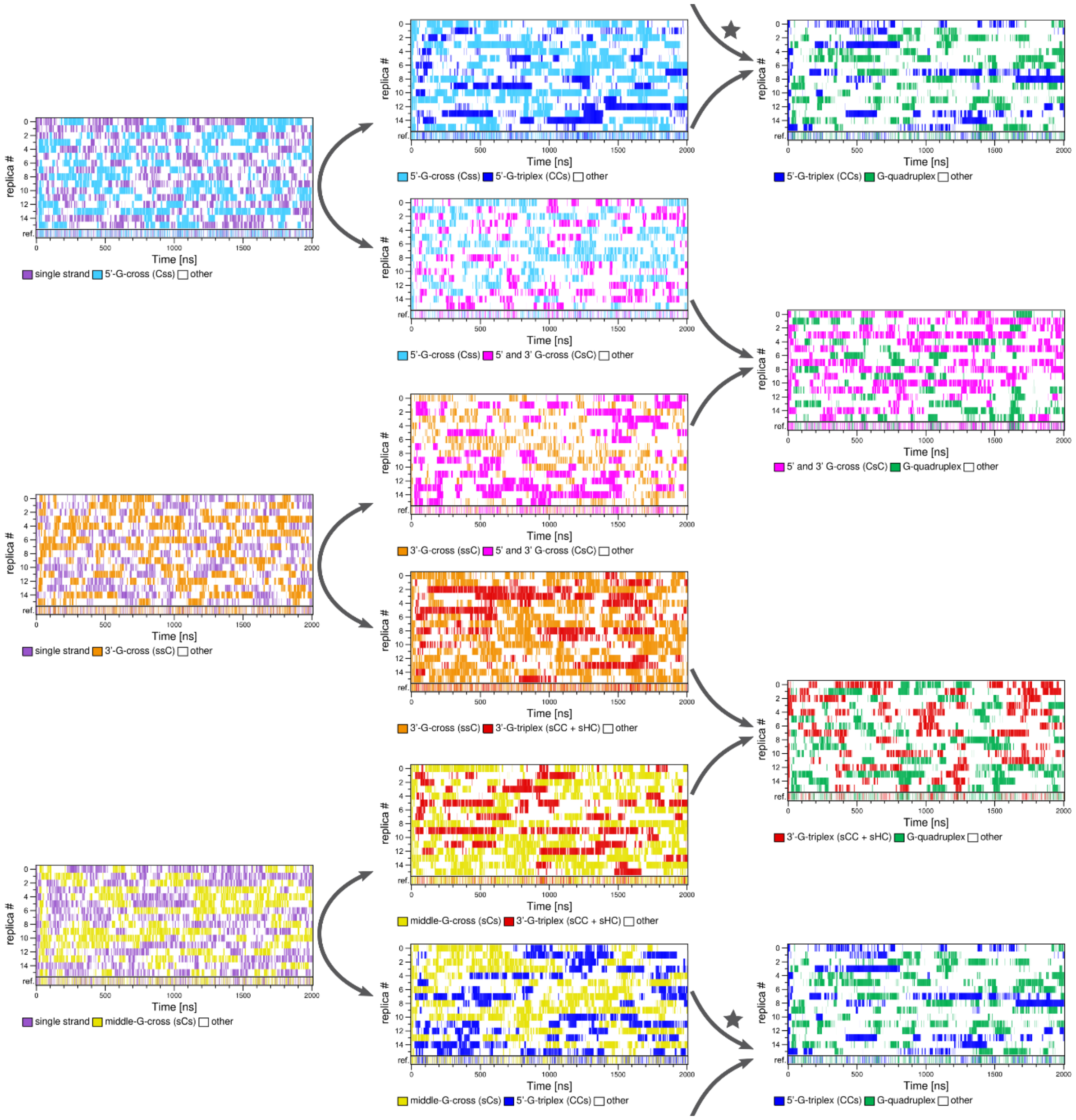

Figure S26 - The panels display sampling of the major conformations defined in Table S11 and corresponding to the vertexes of the folding cube (cf. Figure 6A of the main text) across all 16 continuous (demultiplexed) replicas and reference replica (298 K) and for each of the twelve independent ST-MetaD simulations of the 15-mer (corresponding to the edges of the folding cube). The arrows indicate the relationship of the individual ST-MetaD simulations within the folding cube.

Table S11 – Definition of the major conformations for the 15-mer ST-MetaD simulations corresponding to the beginning and end of the transition path defined by pathCV.

**Transition**	**conf1**	**pathCV**		**conf2**	**pathCV**	
**from→to**		**min**	**max**		**min**	**max**
sss→Css	sss	1.5	10	Css	32	41.5
sss→sCs	sss	1.5	10	sCs	34.5	41.5
sss→ssC	sss	1.5	8	ssC	33.5	41.5
Css→CCs	Css	1.5	11.5	CCs	35.5	41.5
Css→CsC	Css	1.5	7.5	CsC	36.5	41.5
sCs→-CCs	sCs	1.5	10.5	CCs	32.5	41.5
sCs→sHC	sCs	1.5	12.5	sHC	33.5	41.5
CCs→HHH	CCs	1.5	6.5	HHH	36.5	41.5
CsC→HHH	CsC	1.5	9.5	HHH	37.5	41.5
sHC→HHH	sHC	1.5	8.5	HHH	36.5	41.5
ssC→CsC	ssC	1.5	5.5	CsC	33.5	41.5
ssC→sHC	ssC	1.5	12.5	sHC	33.5	41.5

References

1. Case, D. A. B.-S., I. Y.; Brozell, S. R.; Cerutti, D. S.; Cheatham, T. E.; Cruzeiro, V. W. D.; Darden, T. A.; Duke, R. E.; Ghoreishi, D.; Gilson, M. K., et al. *AMBER 2018*, University of Carolina: San Francisco: **2018**.
2. Pronk, S.; Pall, S.; Schulz, R.; Larsson, P.; Bjelkmar, P.; Apostolov, R.; Shirts, M. R.; Smith, J. C.; Kasson, P. M.; van der Spoel, D., et al. GROMACS 4.5: a high-throughput and highly parallel open source molecular simulation toolkit. *Bioinformatics* **2013,** *29*, 845-854.
3. Van Der Spoel, D.; Lindahl, E.; Hess, B.; Groenhof, G.; Mark, A. E.; Berendsen, H. J. GROMACS: fast, flexible, and free. *J Comput Chem* **2005,** *26*, 1701-1718.
4. Hopkins, C. W.; Le Grand, S.; Walker, R. C.; Roitberg, A. E. Long-Time-Step Molecular Dynamics through Hydrogen Mass Repartitioning. *J Chem Theory Comput* **2015,** *11*, 1864-1874.
5. Berendsen, H. J. C.; Postma, J. P. M.; van Gunsteren, W. F.; DiNola, A.; Haak, J. R. Molecular dynamics with coupling to an external bath. *J. Chem. Phys.* **1984,** 81, 3684–3690
6. Ryckaert, JP.; Ciccotti, G.; Berendsen, H. J. C. Numerical integration of the cartesian equations of motion of a system with constraints: molecular dynamics of n-alkanes. *J Comput Phys*, **1977**, 23 (3): 327–341
7. Hopkins, CH. W.; Le Grand, S.; Walker, R. C.; Roitberg, A. E. Long-Time-Step Molecular Dynamics through Hydrogen Mass Repartitioning. *J. Chem. Theory Comput.* **2015**, 11, 4, 1864–1874
8. Flyvbjerg, H.; Petersen, H. G. Error estimates on averages of correlated data. *J. Chem. Phys.* **1989,** 91, 461–466
9. Spiwok, V.; Králová, B. Metadynamics in the conformational space nonlinearly dimensionally reduced by Isomap. *J. Chem. Phys.*, **2011**, 135(22):224504
10. Spiwok, V; Oborsky, P.; Pazúriková, J; Křenek, A.; Králová, B. Nonlinear vs Linear biasing Trp-cage folding simulations, *J. Chem. Phys.* **2015**, 142, 115101

11. Stadbauer, P.; Kührová, P.; Vicherek, L.; Banáš, P.; Otyepka, M.; Trantírek, L.; Šponer, J. Parallel G-triplexes and G-hairpins as potential transitory ensembles in the folding of parallel-stranded DNA G-Quadruplexes. *Nucleic Acid. Res.*, **2019,** 47, 14, 7276–7293,
